# First Synthesis and Characterization of CH_4_@C_60_

**DOI:** 10.1002/anie.201900983

**Published:** 2019-03-12

**Authors:** Sally Bloodworth, Gabriela Sitinova, Shamim Alom, Sara Vidal, George R. Bacanu, Stuart J. Elliott, Mark E. Light, Julie M. Herniman, G. John Langley, Malcolm H. Levitt, Richard J. Whitby

**Affiliations:** ^1^ Chemistry, Faculty of Engineering and Physical Sciences University of Southampton Southampton SO17 1BJ UK; ^2^ Current address: Centre de Résonance Magnétique Nucléaire à Très Hauts Champs FRE 2034 Université de Lyon CNRS Université Claude Bernard Lyon 1 ENS de Lyon 5 Rue de la Doua 69100 Villeurbanne France

**Keywords:** endohedral fullerene, mass spectrometry, NMR spectroscopy, synthetic methods, X-ray diffraction

## Abstract

The endohedral fullerene CH_4_@C_60_, in which each C_60_ fullerene cage encapsulates a single methane molecule, has been synthesized for the first time. Methane is the first organic molecule, as well as the largest, to have been encapsulated in C_60_ to date. The key orifice contraction step, a photochemical desulfinylation of an open fullerene, was completed, even though it is inhibited by the endohedral molecule. The crystal structure of the nickel(II) octaethylporphyrin/ benzene solvate shows no significant distortion of the carbon cage, relative to the C_60_ analogue, and shows the methane hydrogens as a shell of electron density around the central carbon, indicative of the quantum nature of the methane. The ^1^H spin‐lattice relaxation times (*T*_1_) for endohedral methane are similar to those observed in the gas phase, indicating that methane is freely rotating inside the C_60_ cage. The synthesis of CH_4_@C_60_ opens a route to endofullerenes incorporating large guest molecules and atoms.

Soon after the discovery of C_60_ in 1985,^1^ came recognition that its approximately spherical 3.7 Å diameter cavity provides a unique environment in which to isolate single atoms.^2^ Since then endohedral fullerenes, that is, compounds denoted A@C_60_ in which molecules or atoms are enclosed within the fullerene cage, have been the focus of substantial experimental and theoretical efforts.^3^, ^4^, ^5^ Endohedral fullerenes may be synthesized by forming the fullerene in the presence of the endohedral species (particularly successful for metallofullerenes),^3^, ^5^ by high temperature and pressure treatment of the fullerene with the endohedral species (inert gas@C_60_),^6^, ^7^ or by ion bombardment of the fullerene (N@C_60_),^8^ but all give very low incorporation and require extensive purification. Furthermore, these methods are not applicable to the incorporation of small organic molecules.

The macroscopic‐scale preparation of endohedral fullerenes by multi‐step “molecular surgery”^9^, ^10^, ^11^, ^12^ involves chemically opening an orifice in the fullerene, of a size suitable to allow entry of the single molecule. Suturing of this orifice to restore the pristine carbon cage was pioneered by Komatsu^13^, ^14^ and Murata^15^ who reported the first syntheses of H_2_@C_60_ and H_2_O@C_60_ following insertion of H_2_ or H_2_O under high‐pressure, into open‐cage fullerenes **1** and **2**, respectively. Optimized procedures for the synthesis of H_2_@C_60_ and H_2_O@C_60_ have subsequently been reported by ourselves,^16^ based on Murata's open‐cage C_60_ derivative **2**, and also applied to the synthesis of HF@C_60_ (Figure 1).^17^, ^18^

**Figure 1:**
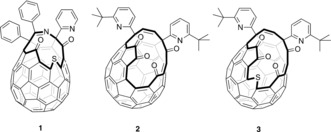
Open‐cage fullerenes. Preparation of H_2_@C_60_ from **1**, and of H_2_O@C_60_ and HF@C_60_ from **2**, is known; as are a series of open‐cage derivatives A@**3**.

The macroscopic quantities of endohedral fullerenes provided by molecular surgery have allowed detailed investigation of physical properties, including by neutron scattering, infrared spectroscopy, and NMR spectroscopy.^19^ These methods have shown that, as a result of the inert and highly symmetrical environment of the cavity, an entrapped molecule behaves much as would be expected in the very low‐pressure gas state,^17^, ^19^, ^20^, ^21^, ^22^, ^23^ displaying free rotation at cryogenic temperatures.^19^, ^20^, ^21^, ^22^, ^23^, ^24^

The 16‐membered orifice of **2** is too small to allow entry of bigger guests, but these can be accommodated by the larger (17‐membered) opening of fullerene **3**.^25^ Insertion of N_2_ and CO_2_,^26^ CH_3_OH and H_2_CO,^27^ CH_4_ and NH_3_,^28^ NO ,^29^ and O_2_,^30^ into **3** have all been recently described, but a procedure for suturing the opening of A@**3** to give A@C_60_ has not yet been reported. In this article, we describe the successful closure of A@**3** to give A@C_60_.

The endohedral fullerenes H_2_@C_60_ and H_2_O@C_60_ are exceptional platforms for the study of nuclear spin isomerism,^24^, ^31^, ^32^, ^33^ in which only certain combinations of nuclear spin states and molecular rotational states are allowed by the Pauli principle. We are particularly interested in CH_4_@C_60_, since spin isomerism is also exhibited by methane, which exists as three nuclear spin isomers with the *J*=0 rotational state having nuclear spin *I*=2, the *J*=1 rotational state having nuclear spin *I*=1, and the *J*=2 rotational state having nuclear spin states *I*=0 and *I*=1.^34^, ^35^ Methane is one of the largest possible guests for C_60_ 36 and herein, we report conditions for optimized CH_4_ encapsulation by **3** and the first successful closure sequence to reform the pristine C_60_ cage. Our work constitutes the first synthesis of CH_4_@C_60_ and raises the exciting prospect of accessing other endohedral fullerenes, A@C_60_, in which the endohedral species is a “large” guest molecule; including A=O_2_, N_2_, CO, NO, NH_3_, CH_3_OH, CH_2_O, and CO_2_, as well as the atoms Ar and Kr.

CH_4_@C_60_ was prepared according to the procedures shown in Scheme 1. Open‐cage fullerene **3** was obtained from bis(hemiketal) **4** 15 according to the published method.^25^ We have previously shown the 17‐membered orifice of **3** to be suitable for entry of a single molecule of methane, achieving 65 % encapsulation by heating **3** at 200 °C under 153 atm of methane.^28^ Upon increasing the pressure of methane above 1500 atm, we obtained CH_4_@**3** with more than 95 % encapsulation of methane (estimated from the ^1^H NMR spectrum) after 22 h at 190 °C. Oxidation with dimethyldioxirane^37^ gave the sulfoxide CH_4_@**5** cleanly. Photochemical removal of the sulfinyl group (SO) has been reported for ring‐contraction of the sulfoxide derivative of open‐cage fullerene **1**, using visible‐light irradiation.^13^, ^38^, ^39^, ^40^ Unfortunately, Murata and co‐workers found that the sulfoxide derivative of **3** (i.e. **5**) does not undergo simple loss of SO under the same conditions, but undergoes decomposition accompanied by a low‐yielding rearrangement to a lactone side product.^41^ However, we noted that the dominant species in the positive‐ion atmospheric pressure photoionization (APPI) mass spectrum of **5** appears at *m*/*z*=1102.18 and corresponds to the radical cation C_82_H_26_N_2_O_4_^+·^ resulting from loss of SO from **5**, indicating that ring‐contraction by photochemical removal of SO is feasible. Since we found that the expected product **2** from the photochemical ring‐contraction is unstable under visible light irradiation, we considered that the reaction might be facilitated if **2** could be trapped in situ as the bis(hemiketal) **4**. We were pleased to observe that in a mixed solvent system of toluene, acetonitrile, and acetic acid (10 % *v*/*v* aq.), irradiation of sulfoxide **5** (containing endohedral water under the partly aqueous reaction conditions), in the visible range for 24 h with an 11 W bulb, gave a mixture of **4** and H_2_O@**4** in 25 % yield of isolated product, with a similar amount of unreacted **5** remaining. A longer period of irradiation did not lead to a higher yield of **4**. Product(s) of polymerization or decomposition, which were not identified, accounted for the remaining material, and none of the lactone product recovered by Murata et al. was isolated.

**Scheme 1:**
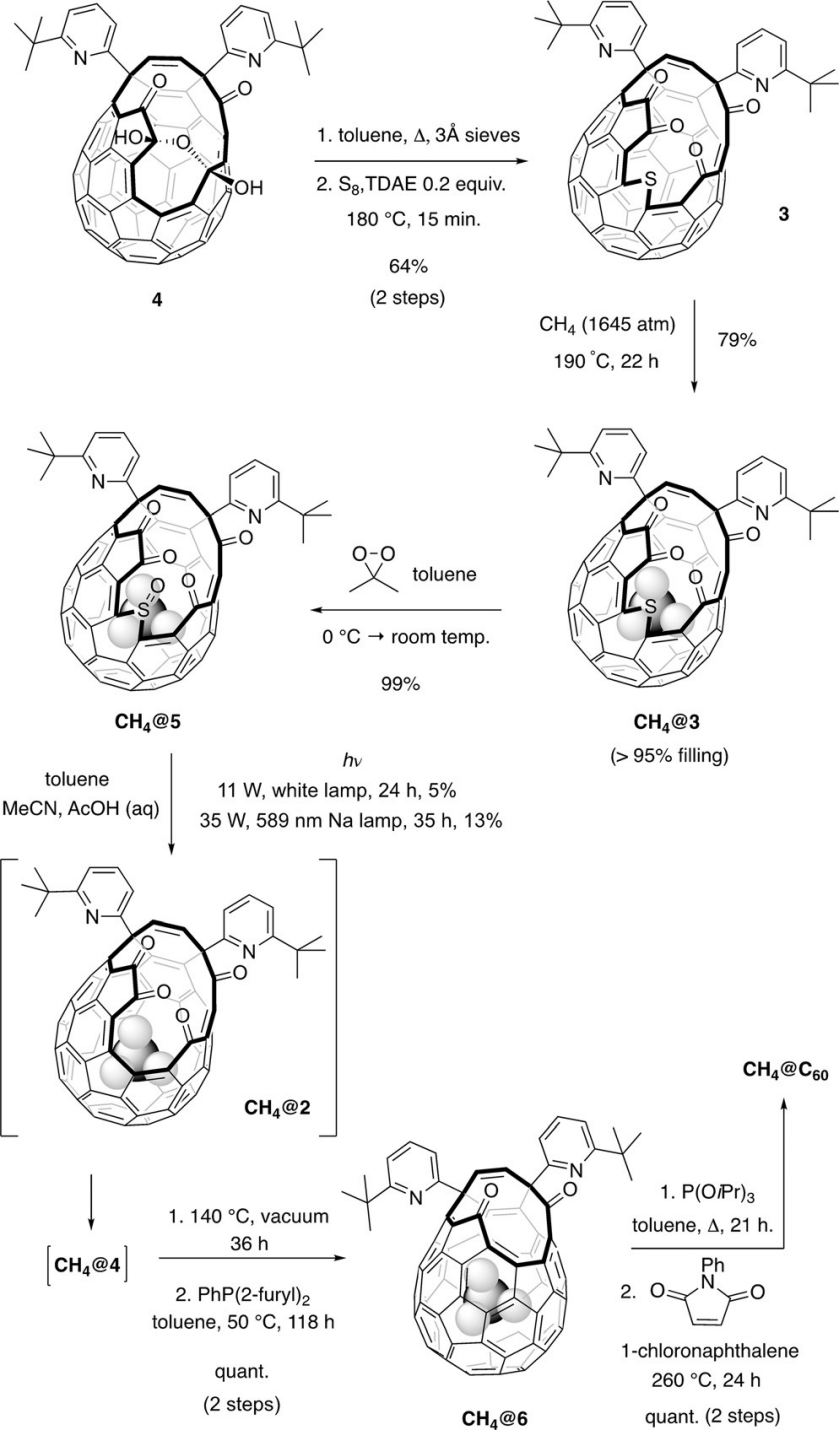
Synthesis of CH_4_@C_60_. Optimized CH_4_ encapsulation by **3** and a successful closure sequence, involving photochemical desulfinylation, are applied to the first synthesis of CH_4_@C_60_.

When CH_4_@**5** was subjected to identical photochemical conditions, the corresponding product of SO loss followed by hydration, CH_4_@**4**, was obtained in only 5 % yield, retaining more than 95 % methane filling. We confirmed that the observed drop in yield is due to the presence of endohedral methane by carrying out photolysis on a sample of CH_4_@**5** with 83 % filling (Supporting Information, Section S5), from which the product CH_4_@**4** was obtained with only 57 % filling as a result of the much higher‐yielding conversion of the portion of the material that does not contain methane.

The yield of the photochemical ring‐contraction was significantly increased upon switching to irradiation with monochromatic (yellow) light at 589 nm, using a low‐pressure sodium lamp. A mixture of **5** and H_2_O@**5** was converted to the bis(hemiketal) mixture (**4** + H_2_O@**4**) in 43 % yield of isolated product. The corresponding reaction of CH_4_@**5** under irradiation at 589 nm gave CH_4_@**4** in a yield of 13 %, in accordance with the expected inhibition of the reaction by endohedral methane, and is a valuable improvement in comparison with the very low yield obtained using white light. It is rare for endohedral species to affect the reactivity of the fullerene cage,^42^, ^43^ particularly in such a dramatic (and unfortunate) fashion, but while it is disappointing that this step remains low‐yielding, with CH_4_@**4** in hand we were now able to adapt known procedures for suturing of the bis(hemiketal) orifice to an intact C_60_ shell.

CH_4_@**4** (more than 95 % filling) was contaminated by a trace of H_2_O@**4**, identified by the ^1^H NMR resonance of endohedral water at *δ*=−9.84 ppm^16^ and distinct from the ^1^H resonance for endohedral methane in CH_4_@**4**, which appears as a sharp singlet at *δ*=−11.22 ppm (CDCl_3_). Since the percentage filling of H_2_O will be amplified by a factor of approximately five during photochemical ring contraction (Supporting Information, Section S5.1), we extrapolate the methane filling in CH_4_@**5** to be more than 99.5 %. To avoid final contamination of CH_4_@C_60_ by H_2_O@C_60_, CH_4_@**4** was heated at 140 °C under a dynamic vacuum (approximately 0.5 mm Hg) for 36 h to obtain CH_4_@**2** with accompanying removal of the endohedral water contaminant. No loss of CH_4_ was observed. Subsequent reduction to CH_4_@**6** using di‐(2‐furyl)phenylphosphine in toluene, at a temperature of 50 °C (too low for water re‐entry), gave CH_4_@**6** (more than 95 % filling) in quantitative yield. Endohedral methane appears as a singlet with a shift of *δ*_H_=−9.82 ppm (700 MHz, [D_8_]THF, 295 K) in the ^1^H NMR spectrum of CH_4_@**6**, and no H_2_O@**6** was present. Finally, the orifice of CH_4_@**6** was sutured, using identical conditions to those reported for H_2_O@**6**,^16^ and CH_4_@C_60_ was obtained with 100.0±0.3 % filling after removal of traces of (empty) C_60_ by preparative HPLC on a Cosmosil™ Buckyprep column. An independently prepared sample of H_2_O@C_60_ was found to co‐elute with CH_4_@C_60_, confirming the necessity for removal of contaminant endohedral water earlier in the synthesis.

The positive‐ion APPI mass spectrum of CH_4_@C_60_ is in agreement with the calculated isotope distribution pattern for C_61_H_4_ (Figure 2), and the ultrahigh resolution also confirms that H_2_O@C_60_ is not present since the isotope patterns for CH_4_@C_60_ and H_2_O@C_60_ were shown to be non‐overlapping (Supporting Information, Section S4).

**Figure 2:**
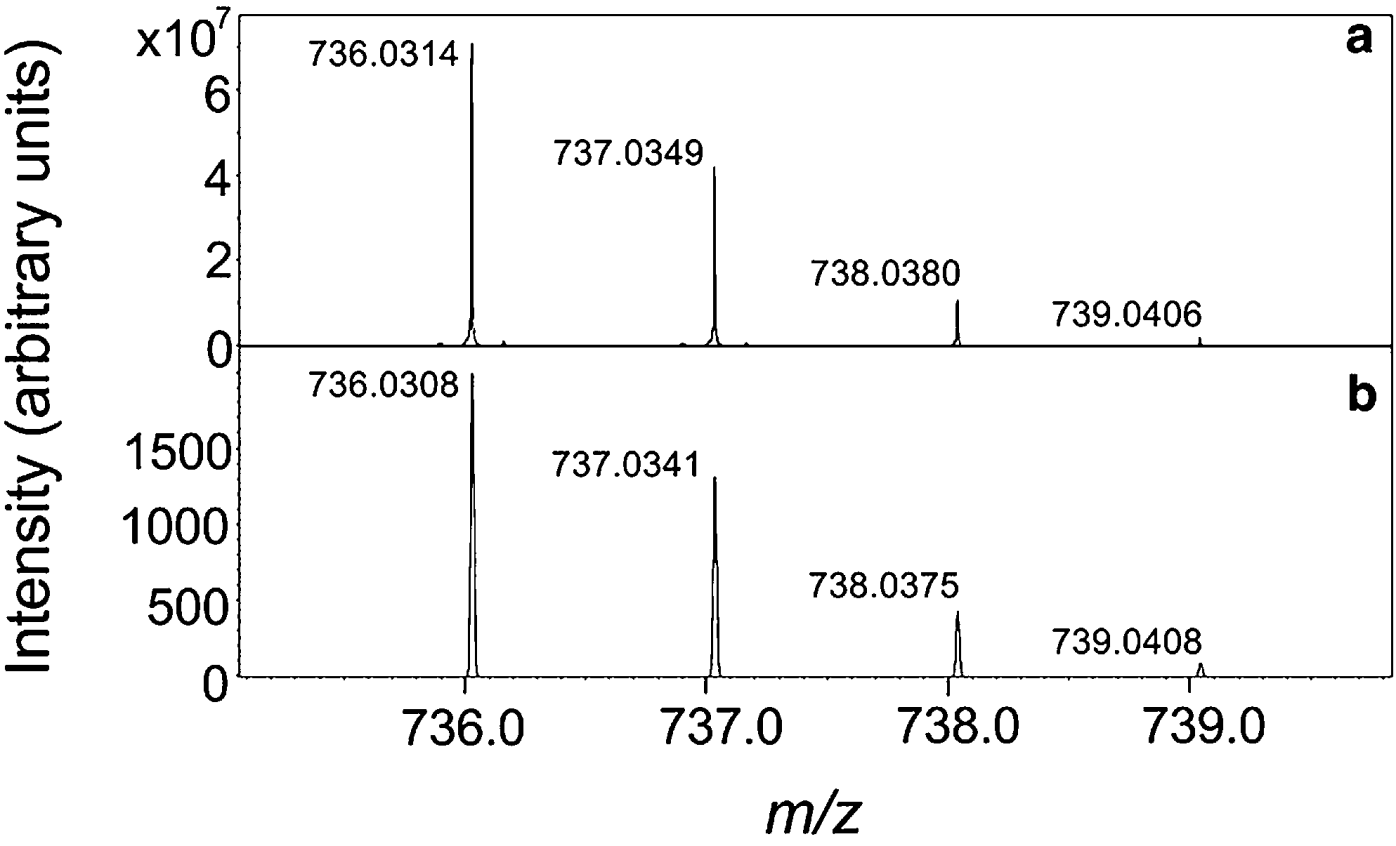
Positive‐ion APPI mass spectrum of CH_4_@C_60_. a) Experimental data and b) model isotope pattern for C_61_H_4_; *m*/*z* 735–740.

A crystal structure of the nickel(II) octaethylporphyrin/ benzene solvate^44^ of CH_4_@C_60_ was obtained (CCDC 1858399 contain the supplementary crystallographic data for this paper. These data can be obtained free of charge from The Cambridge Crystallographic Data Centre.) and is similar to that reported for the equivalent C_60_ solvate,^45^ with the exception of a spherically symmetrical electron density distribution located at the center of the fullerene, corresponding to the endohedral methane molecule. The electron density map shows a faint spherical shell around the main center of the endohedral electron density, at a radius of 1.03 Å (Figure 3). This shell of distributed electron density corresponds to the delocalized nuclear wavefunction of the methane hydrogens, as expected for a quantum description of the freely rotating molecule. This quantum description is well‐established for the analogous systems H_2_@C_60_, H_2_O@C_60_, and HF@C_60_, which have been extensively studied by neutron‐scattering and infrared spectroscopy.^17^, ^20^, ^21^, ^22^ A classical description in which the methane explores a random set of orientations would give a similar result. There is no geometrical evidence (within 3‐sigma) for distortion of the cage relative to the C_60_ analogue, or displacement of the methane from its center.

**Figure 3:**
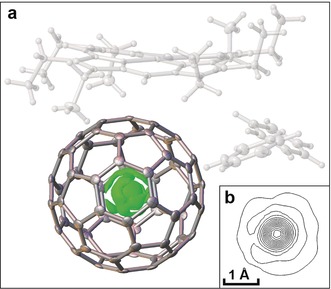
Crystal structure for the nickel(II) octaethylporphyrin/ benzene solvate of CH_4_@C_60_. a) Thermal ellipsoids for the cage atoms of CH_4_@C_60_ and the difference electron density map for endohedral CH_4_ (surface drawn at the 0.6 e Å^3^ level) are shown. Ni^II^OEP and benzene are shown as thermal ellipsoids in white and all thermal ellipsoids are shown at 50 % probability. b) Selected slice through the center of difference electron density at the CH_4_ position, contours drawn at approximately 0.9 e Å^3^. A faint shell of electron density at a radius of 1.03 Å from the center of the cage is visible. This corresponds to the delocalized wavefunction of the methane hydrogen atoms. CCDC 1858399 contain the supplementary crystallographic data for this paper. These data can be obtained free of charge from The Cambridge Crystallographic Data Centre. Structure details are reported in Section S6 of the Supporting Information.

Detailed NMR characterization of CH_4_@C_60_ was carried out. The ^1^H NMR spectrum in 1,2‐dichlorobenzene‐*d*_4_ displays a singlet at *δ*_H_=−5.71 ppm, where the shift results from the shielding effect of the C_60_ cage, compared with ^12^CH_4_ in the gas phase, which has a chemical shift of *δ*_H_=2.166±0.002 ppm.^46^ From the natural abundance ^13^CH_4_@C_60_, the measured coupling is ^1^ *J*_HC_=124.3±0.2 Hz (at 295 K), in comparison with ^1^ *J*_HC_=125.3 Hz (at 292 K) measured in the gas phase.^47^ The liquid state ^13^C{^1^H} NMR spectrum reports a sharp singlet for endohedral methane at *δ*_C_=−13.63 ppm in 1,2‐dichlorobenzene‐*d*_4_, again shielded in comparison with the reported shift of *δ*_C_=−8.648±0.001 ppm measured in the gas phase^46^ (Figure 4 a,b).

**Figure 4:**
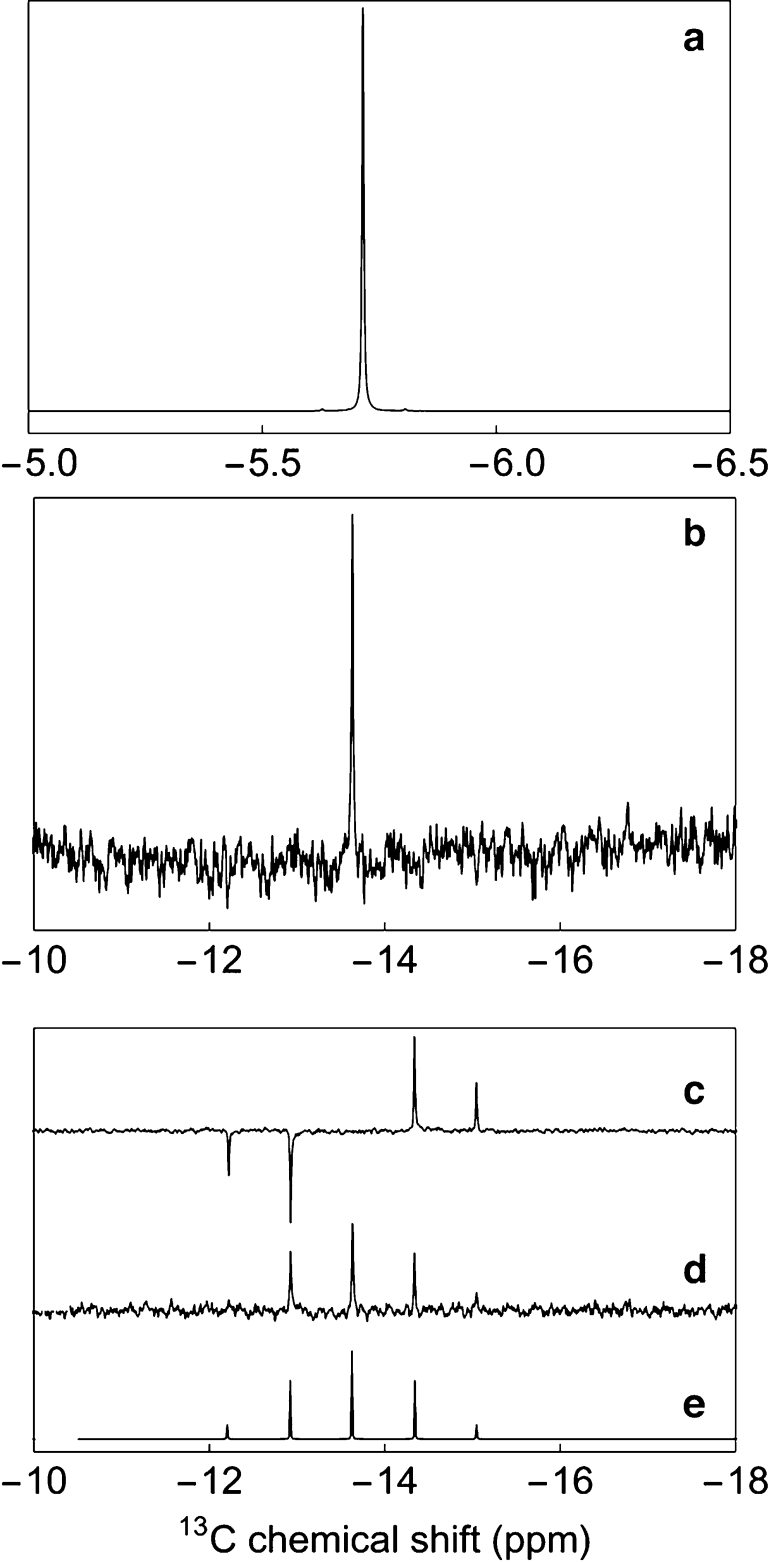
^1^H and ^13^C NMR resonances for endohedral methane in CH_4_@C_60_. a) Experimental ^1^H NMR resonance of CH_4_@C_60_ acquired with 1 transient, b) Experimental ^13^C NMR resonance of CH_4_@C_60_ with ^1^H WALTZ16 decoupling (nutation frequency=14.2 kHz), acquired with 4928 transients and a delay of 10 s between scans, c) Experimental non‐proton‐decoupled ^13^C INEPT spectrum, acquired with 35 840 transients and a delay of 4.5 s between scans, d) Experimental non‐proton‐decoupled ^13^C NMR spectrum excited by a single 90° pulse, acquired with 35 840 transients and a delay of 4.5 s between scans, e) Numerical simulation of (d) using *SpinDynamica*.^50^ All experimental spectra were acquired for a degassed 4.5 mm sample of CH_4_@C_60_ in 1,2‐dichlorobenzene‐*d*_4_ at 16.45 T (^1^H nuclear Larmor frequency=700 MHz and ^13^C nuclear Larmor frequency=176 MHz) and 295 K.

Figure 4 c,d shows the relevant section of the INEPT NMR spectrum of ^13^ CH_4_@C_60_, alongside experimental and simulated proton‐coupled ^13^C NMR spectra. The INEPT pulse sequence was used as defined by Morris and Freeman^48^ with an interpulse delay of *τ*=$\frac{1}{4\;J_{HC}}$ =2.012 ms (*J*_HC_=124.3 Hz). The experimental ^13^C resonance is a 1:4:6:4:1 quintet with chemical shift *δ*_C_=−13.63 ppm. The ^13^C NMR resonance for the cage in CH_4_@C_60_ appears at *δ*_C_=143.20 ppm, shifted by Δ*δ*=+0.52 ppm relative to C_60_ itself. This is a large deshielded shift of the cage ^13^C NMR resonance in comparison with the effect of smaller molecular endohedral species (HF@C_60_, Δ*δ*=+0.04 ppm,^17^ H_2_@C_60_, Δ*δ*=+0.08 ppm,^16^ and H_2_O@C_60_, Δ*δ*=+0.11 ppm^15^, ^16^), consistent with the large size of methane. Correlation of Δ*δ* with the van der Waals radius of the enclosed species has been described for the inert gas@C_60_ series.^49^

The ^1^H spin‐lattice relaxation time constant of ^12^CH_4_@C_60_ was found to be *T*_1_=1.4904±0.0005 s at 295 K. Measurement of *T*_1_ as a function of temperature indicates a clear increase in relaxation rate constant (*T*_1_^−1^) with increasing temperature. This is indicative of a significant spin‐rotation contribution to the relaxation, and is consistent with ^1^H relaxation of methane in the gas phase^51^ (Figure 5 a).

**Figure 5:**
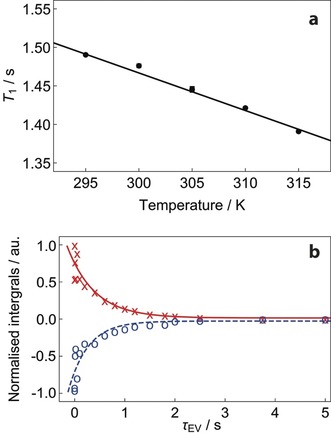
Experimental ^1^H and ^13^C spin‐lattice relaxation times for endohedral methane in CH_4_@C_60_. a) Experimental ^1^H spin lattice relaxation as a function of temperature for ^12^CH_4_@C_60_. The best straight‐line fit to the experimental data points is shown. ^1^H longitudinal relaxation times were measured using the inversion‐recovery pulse sequence; b) Experimental ^13^C spin‐lattice relaxation curves for ^13^CH_4_@C_60_ (natural abundance). Spectra were acquired for a degassed 4.5 mm solution of CH_4_@C_60_ in 1,2‐dichlorobenzene‐*d*_4_ at 16.45 T (^1^H nuclear Larmor frequency=700 MHz) and 295 K. Red data points ***x*** correspond to the satellite at *δ*=−5.638 ppm; Blue data points **o** correspond to the satellite at *δ*=−5.815 ppm. The ^13^C longitudinal relaxation time *T*_1_ was measured using the pulse sequence described in Section S3.2 of the Supporting Information. All signal amplitudes were normalized to the maximum integral (second data point, *τ*_EV_=1 ms). The fitted curves have single exponential form.

The ^13^C *T*_1_ values for endohedral methane, reported by the ^13^C satellites of the ^1^H spectrum using a modified INEPT sequence (Supporting Information, Section S3.2), are slightly different: *T*_1_=0.39±0.14 s for the less shielded satellite, and *T*_1_=0.55±0.14 s for the more shielded satellite (Figure 5 b). This difference is likely to be associated with cross‐correlated relaxation effects.^52^

In summary, CH_4_@C_60_, the first example of an organic molecule trapped in C_60_, has been synthesized. CH_4_ is the largest molecule, with the greatest number of atoms, to have been encapsulated in C_60_ to date. The first step of the orifice contraction was strongly inhibited by the presence of endohedral methane, resulting in a low yield for the key photolytic step. CH_4_@C_60_ was characterized by high resolution mass spectrometry, NMR spectroscopy, and X‐ray crystallography. ^1^H spin‐lattice relaxation times for endohedral methane are similar to those observed in the gas phase, providing evidence that methane is freely rotating inside the C_60_ cage. The experimental ^13^C NMR chemical shift of the cage carbon is shifted by +0.52 ppm relative to empty C_60_. We find no evidence for distortion of the cage from a crystal structure of the nickel(II) octaethylporphyrin/ benzene solvate of CH_4_@C_60_. In the crystal structure, the hydrogen atoms of methane appear as a spherically symmetric sphere of electron density, consistent with a delocalized quantum state. Neutron scattering, infrared spectroscopy, and cryogenic NMR spectroscopy experiments are now planned to study spin‐isomerism and spin‐isomer conversion of the encapsulated methane molecules. The successful synthesis of CH_4_@C_60_ opens a route to novel endofullerenes A@C_60_ enclosing “large” endohedral species A, such as A=O_2_, NO, NH_3_, N_2_, CO_2_, CH_3_OH, and H_2_CO, with exciting prospects for the study of these encapsulated small molecules.

## Experimental Section

Details of the synthesis and characterization of CH_4_@C_60_ are in the Supporting Information. Original data may be found at https://doi.org/10.5258/SOTON/D0809.

## Conflict of interest

The authors declare no conflict of interest.

## Supporting information

As a service to our authors and readers, this journal provides supporting information supplied by the authors. Such materials are peer reviewed and may be re‐organized for online delivery, but are not copy‐edited or typeset. Technical support issues arising from supporting information (other than missing files) should be addressed to the authors.

Supplementary
