# Supplementary material for: First Synthesis and Characterization of CH4@C60
Source: Angew Chem Int Ed Engl. 2019 Mar 12;58(15):5038–43. doi: 10.1002/anie.201900983 (PMC6492075; doi:10.1002/anie.201900983)
Supplement: Supplementary file 1 — Supplementary [file ANIE-58-5038-s001.pdf]

Angewandte Chemie
Eine Zeitschrift der Gesellschaft Deutscher Chemiker

Supporting Information

First Synthesis and Characterization of $CH_4@C_{60}$

*Sally Bloodworth, Gabriela Sitinova, Shamim Alom, Sara Vidal, George R. Bacanu, Stuart J. Elliott, Mark E. Light, Julie M. Herniman, G. John Langley, Malcolm H. Levitt, and Richard J. Whitby**

anie_201900983_sm_miscellaneous_information.pdf

Author Contributions

S.B. Data curation: Lead; Investigation: Lead; Methodology: Lead; Writing—original draft: Lead; Writing—review & editing: Lead
G.S. Investigation: Supporting
S.A. Investigation: Supporting
S.V. Investigation: Supporting
G.B. Formal analysis: Equal; Investigation: Supporting; Methodology: Equal; Writing—original draft: Supporting
S.E. Formal analysis: Supporting; Investigation: Supporting; Methodology: Equal; Supervision: Supporting; Writing—review & editing: Supporting
M.L. Data curation: Lead; Formal analysis: Lead; Writing—original draft: Supporting
J.H. Investigation: Supporting; Methodology: Supporting
G.L. Methodology: Supporting; Supervision: Supporting; Writing—review & editing: Supporting
M.L. Conceptualization: Equal; Funding acquisition: Equal; Project administration: Supporting; Supervision: Equal; Writing—original draft: Supporting; Writing—review & editing: Supporting
R.W. Conceptualization: Lead; Funding acquisition: Equal; Project administration: Lead; Supervision: Lead; Writing—original draft: Supporting; Writing—review & editing: Supporting.

CONTENTS

		Page
S1.	Experimental procedures and characterisation data	S3
	S1.1 General methods	S3
	S1.2 CH_4@**3**	S4
	S1.3 CH_4@**5**	S4
	S1.4 CH_4@**6**	S5
	S1.5 CH_4@C_{60}	S7
S2.	^{13}C NMR simulation using *SpinDynamica*	S9
S3.	Measurement of spin-lattice relaxation (T_1)	S10
	S3.1 Experimental ^{1}H spin-lattice relaxation curves	S10
	S3.2 ^{13}C spin-lattice relaxation measurement using INEPT	S11
S4.	Mass spectrometry	S12
S5.	Measurement of the relative yields for photochemical closure of CH_4@**5** *vs.* H_2O@**5**	S12
	S5.1 Experimental method	S13
S6.	X-Ray structure determination of CH_4@C_{60}	S14
	S6.1 Experimental method	S15
	S6.2 Crystal data	S16
	S6.3 Structure quality indicators	S16
	S6.4 Generated precession images	S17
	S6.5 Data plots: diffraction data	S18
	S6.6 Data plots: refinement and data	S18
	S6.7 Reflection statistics	S18
S7.	Full references for those abbreviated in the main paper	S28
S8.	References for the supporting information	S28
S9.	Author contributions	S29

S1. Experimental procedures and characterisation data

S1.1 General methods

Reactions were conducted under an argon atmosphere using standard Schlenk and syringe techniques with freshly distilled solvents. All apparatus was dried in a hot oven (>140 °C, 12 h) before being cooled in a sealed desiccator over silica gel or assembled while hot and cooled under vacuum (0.1 mm Hg).

Toluene was freshly distilled from sodium benzophenone ketal under argon. Technical grade 1-chloronaphthalene (≥85%) was distilled under nitrogen and solutions in 1-chloronaphthalene were degassed under reduced pressure (<1 mm Hg) until evolution of gases had ceased. Triisopropyl phosphite was distilled over sodium at reduced pressure. Dimethyldioxirane[1] and di-(2-furyl)phenylphosphine[2] were prepared according to the published procedures. All other reagents, solvents or gases were used as received from commercial suppliers.

NMR spectra were recorded on a Bruker AVIIIHD500 FT-NMR spectrometer, or Bruker Ascend 700 NB magnet with Bruker AVANCE NEO console and Bruker TCI prodigy 5 mm liquids cryoprobe; in the indicated solvent at 295K. ^{1}H chemical shifts are reported as values in ppm referenced to residual solvent. ^{1}H NMR spectra collected in 1,2-dichlorobenzene-d_4 are referenced to residual solvent at δ_H = 6.93 ppm; and this solvent chemical shift is referenced to TMS (δ_H = 0.00 ppm). The following abbreviations are used to assign multiplicity and may be compounded: s = singlet, d = doublet, t = triplet, q = quartet and m = multiplet. Coupling constants, J, are measured in Hertz (Hz). ^{13}C NMR spectra were ^{1}H decoupled unless otherwise stated, and are referenced to solvent, including to 1,2-dichlorobenzene-d_4 at δ_C = 132.39 ppm (singlet); the solvent chemical shift is referenced to TMS (δ_C = 0.00 ppm). The confidence limits of the chemical shift are dominated by the chemical shift of reference solvents, reported to 2 d.p, and are larger than the instrumental error (± 0.002 ppm). Chemical shifts are therefore reported to 2 d.p and no instrumental error is stated.

Positive ion atmospheric pressure photoionisation (APPI) mass spectra were recorded using a solariX Fourier transform ion cyclotron resonance mass spectrometer (Bruker Daltonik GmbH, Bremen, Germany) equipped with a 4.7 T actively shielded superconducting magnet and an Infinity cylindrical analyser cell. The sample was diluted using toluene and directly infused into the APPI source with a 100 µL Hamilton syringe at a flow rate of 5 µL/min. The capillary voltage was set at -2000 V, drying temperature of 180 °C, a drying gas flow rate of 4 µL/min and a nebuliser gas pressure of 2 bar. The source was equipped with a 10.6 eV krypton lamp and a vaporisation temperature of 370 °C was utilised. 16 scans were summed over the m/z range 150-1500 with a data set size of 2 M resulting in a resolving power of 200,000 at m/z 400. Data were acquired using Solarixcontrol v 1.5.0 and processed using BrukerDataAnalysis v 4.0. Positive ion electrospray mass spectra were recorded using a MaXis time of flight (TOF) mass spectrometer (Bruker Daltonik GmbH, Bremen, Germany).

Bis(hemiketal) open-fullerene **4**[3] and sulfide open-fullerene **3**[4,5] were prepared according to the published methods.

S1.2 CH_4@3

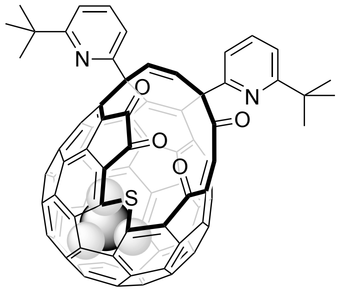

A glass reactor tube was charged with powdered open-fullerene **3** (255 mg, 0.224 mmol) and loosely plugged with glass wool. The tube was inserted into a purpose-built steel housing and flushed with CH_4 (4 × approx. 20 atm) before charging with CH_4 to 997 atm., at room temperature, using a Sitec 750.01 hand-operated pressure intensifying syringe (1000 bar working pressure, 30 mL capacity). The reactor was then heated to 190 °C and maintained at this temperature for 22 h, with a stable internal pressure of 1643 – 1648 atm. during this time, before cooling to room temperature and slow release of the pressure. Purification of the residual solid by column chromatography (SiO_2 eluted with a gradient of toluene → 10% EtOAc in toluene) gave the title compound as a dark red/brown solid (203 mg, 79%). >95% Filling was determined by comparison of integrals in the experimental ^{1}H NMR spectrum and spectroscopic data were consistent with the published data.[5]

S1.3 CH_4@5

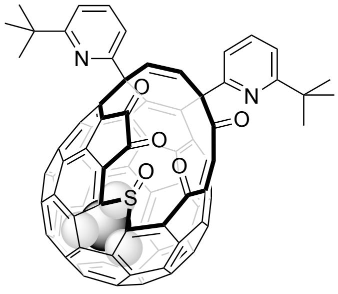

To a stirred solution of CH_4@**3** (350 mg, 0.304 mmol) in toluene (30 mL) at 0 °C, was added dimethyldioxirane (4.64 mL of a 98.2 mM solution in acetone, 0.45 mmol) rapidly using an ice-chilled syringe. The resulting mixture was stirred at 0 °C for 10 min. before removal of the cooling bath and stirring for 1 h, during which time the mixture warmed to room temperature. Solvents were removed *in vacuo* to give the title compound as a crude brown powder (351 mg, 99%) which was used directly in the next step without purification.

^{1}H NMR (500 MHz, THF-d_8) δ = 7.68 (1H, t, J = 7.9 Hz), 7.67 (1H, t, J = 7.9 Hz), 7.34 (1H, dd, J = 7.9, 0.9 Hz), 7.32 (1H, dd, J = 7.9, 0.8 Hz), 7.30 (1H, dd, J = 7.9, 0.8 Hz), 7.27 (1H, dd, J = 7.9, 0.9 Hz), 7.19 (1H, d, J = 10.3 Hz), 6.69 (1H, d, J = 10.3 Hz), 1.20 (9H, s), 1.16 (9H, s), −11.26 (3.8H, s, endohedral CH_4) ppm.

HRMS-ESI+ (*m/z*): Calcd. for $[^{12}C_{83}H_{31}N_2O_5S]^+$, 1167.1948; found 1167.1954. Calcd. for $[^{12}C_{82}{}^{13}CH_{31}N_2O_5S]^+$ isotope, 1168.1981; found 1168.1988. Calcd. for $[^{12}C_{81}{}^{13}C_2H_{31}N_2O_5S]^+$ isotope, 1169.2013; found 1169.2010. Calcd. for $[^{12}C_{80}{}^{13}C_3H_{31}N_2O_5S]^+$ isotope, 1170.2047; found 1170.2025. Calcd. for $[^{12}C_{79}{}^{13}C_4H_{31}N_2O_5S]^+$ isotope, 1171.2082; found 1171.2031.

S1.4 CH_4@**6**

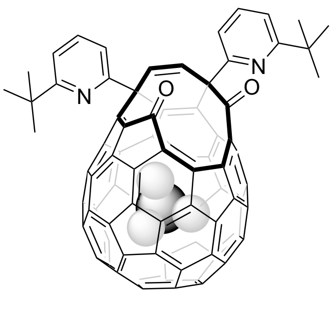

Intermediate CH_4@**4** was obtained using either procedure A or B:

Procedure A: A purpose-built reaction vessel (Figure S1.1) was charged with CH_4@**5** (84.0 mg, 0.0720 mmol) and the apparatus placed under an atmosphere of argon. MeCN (80 mL, degassed), AcOH (16 mL of a degassed 10% *v/v* aqueous solution) and toluene (48 mL) were added and the resulting mixture was vigorously stirred under irradiation with a Pro-Lite™ 11W (800 Lm, 4000 K) lamp, for 24 h, without cooling. Solvents were then removed *in vacuo*. Purification by rapid, repeat column chromatography (SiO_2 eluted with a 90:8:2 mixture of toluene:EtOAc:AcOH; then SiO_2 eluted with 5% AcOH in toluene) gave CH_4@**4** as a black solid (4.0 mg, 5%) which was used directly in the next step.

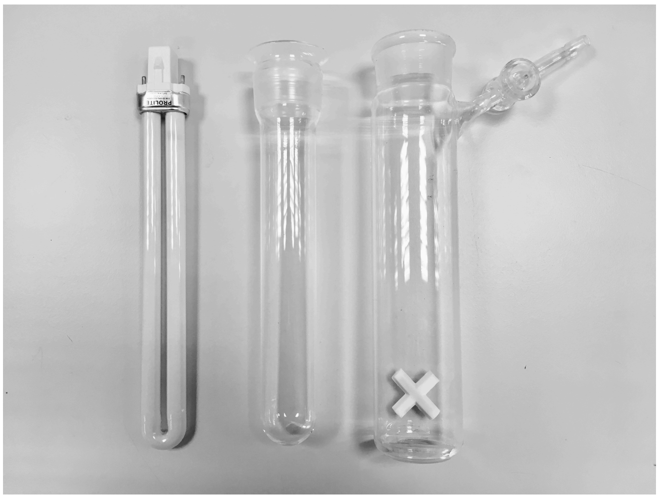

Figure S1.1 Purpose-built photo-reactor for light-induced desulfinylation of CH_4@**5**.

Procedure B: A dry Schlenk flask was charged with CH_4@**5** (52.0 mg, 0.0445 mmol) and the apparatus placed under an atmosphere of argon. MeCN (40 mL, degassed), AcOH (8 mL of a degassed 10% *v/v* aqueous solution) and toluene (15 mL) were added and the resulting mixture was vigorously stirred under irradiation with a low-pressure sodium lamp (35 W) at 10 mm distance, for 35 h, without cooling. Solvents were then removed *in vacuo*. Purification by rapid, repeat column chromatography (SiO_2 eluted with a 90:8:2 mixture of toluene:EtOAc:AcOH; then SiO_2 eluted with toluene → 5% AcOH in toluene) gave CH_4@**4** as a black solid (6.9 mg, 13%) which was used directly in the next step.

CH_4@**4** obtained above (4.0 mg, 0.0035 mmol) was heated at 140 °C under vacuum (1 mm/Hg) for 36 h before cooling to room temperature under vacuum, and dissolution in toluene (1 mL). Di-(2-furyl)phenylphosphine (16.0 mg, 0.066 mmol) was added and the resulting mixture stirred at 50 °C for 118 h with exclusion of light. After cooling to room temperature, solvents were removed *in vacuo*. Purification by column chromatography (SiO_2 eluted with a gradient of 1:1 hexane:toluene → toluene) gave the title compound, CH_4@**6**, as a shiny brown/black solid (3.9 mg, quant. yield from CH_4@**4**). >95% Filling was determined by comparison of integrals in the experimental ^{1}H NMR spectrum. Although this step gave a quantitative measured yield there is likely to be a significant error associated with the very small quantity of material used.

^{1}H NMR (700 MHz, THF-d_8) δ = 7.77 (1H, t, J = 7.8 Hz), 7.74 (1H, t, J = 7.8 Hz), 7.59 (1H, d, J = 7.8 Hz), 7.50 (1H, d, J = 7.8 Hz), 7.33 (1H, d, J = 7.8 Hz), 7.32 (1H, d, J = 7.8 Hz), 7.18 (1H, d, J = 10.1 Hz), 7.15 (1H, d, J = 10.1 Hz), 1.28 (9H, s), 1.18 (9H, s), −9.82 (4H, s, endohedral CH_4) ppm.

$^{13}C\{^{1}H\}$ NMR (176 MHz, THF-d_8) δ = 199.28, 191.70, 169.92, 169.54, 166.53, 163.35, 156.79, 151.35, 150.14, 149.17, 149.08, 148.93, 148.65, 148.60, 148.29, 148.19, 148.12, 147.60, 147.50, 147.31, 147.30, 147.13, 147.12, 147.07, 146.93, 146.79, 146.45, 146.30, 145.95, 145.90, 145.78, 145.58, 145.22, 144.35, 144.32, 144.11, 143.78, 143.75, 143.66, 143.55, 142.55, 142.48, 142.35, 142.22, 141.91, 141.56, 141.09, 140.97, 140.91, 140.62, 140.28, 139.86, 139.46, 138.90, 138.70, 138.66, 138.62, 138.49, 137.72, 136.30, 136.01, 135.19, 134.82, 134.34, 133.35, 132.71, 131.04, 129.84, 129.09, 126.21, 121.49, 120.80, 118.67, 117.97, 62.11, 56.05, 38.65, 38.55, 30.48, −16.54 (endohedral CH_4) ppm.

HRMS-APPI (m/z): Calcd. for $[^{12}C_{83}H_{30}N_2O_2]^{+\bullet}$, 1086.2302; found 1086.2305. Calcd. for $[^{12}C_{82}{}^{13}CH_{30}N_2O_2]^{+\bullet}$ isotope, 1087.2335; found 1087.2329. Calcd. for $[^{12}C_{81}{}^{13}C_2H_{30}N_2O_2]^{+\bullet}$ isotope, 1088.2369; found 1088.2365. Calcd. for $[^{12}C_{80}{}^{13}C_3H_{30}N_2O_2]^{+\bullet}$ isotope, 1089.2402; found 1089.2392. Calcd. for $[^{12}C_{79}{}^{13}C_4H_{30}N_2O_2]^{+\bullet}$ isotope, 090.2436; found 1090.2436.

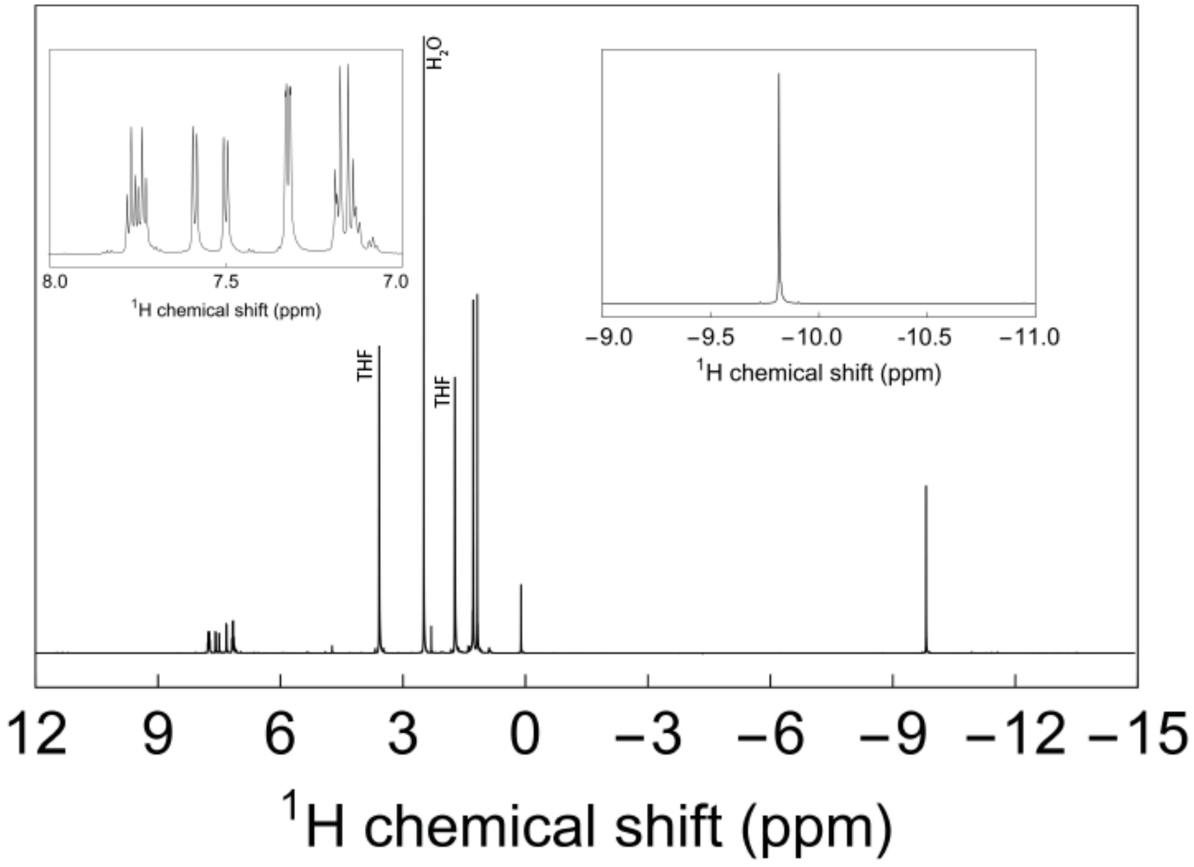

Figure S1.2 Experimental ^{1}H NMR spectrum of CH_4@**6** (1.5 mM in degassed THF-d_8) acquired at 16.45 T (^{1}H nuclear Larmor frequency = 700 MHz) and 295 K with 64 transients and a delay of 45 s between scans. The peak corresponding to $C\underline{H}_4$@**5** is found at −9.82 ppm. The spectrum was processed using Lorentzian line broadening (full-width at half-maximum = 1.75 Hz).

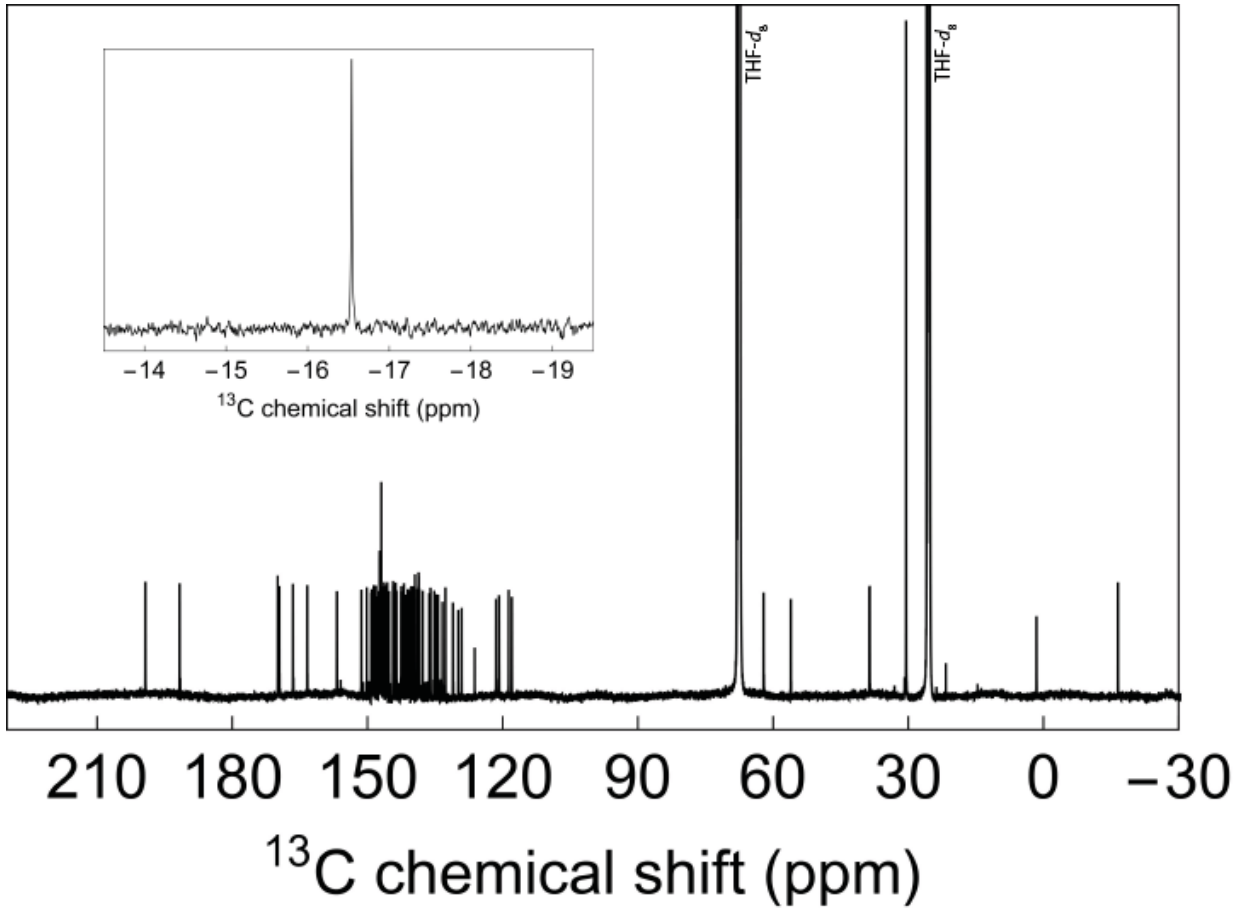

Figure S1.3 Experimental ^{13}C NMR spectrum of CH_4@**6** (1.5 mM in degassed THF-d_8) with ^{1}H WALTZ16 decoupling (nutation frequency = 20 kHz) acquired at 16.45 T (^{13}C nuclear Larmor frequency = 176 MHz) and 295 K with 20000 transients and a delay of 10 s between scans. The peak corresponding to $\underline{C}H_4$@**5** is found at −16.54 ppm and is shown in the expansion. The spectrum was processed using Lorentzian line broadening (full-width at half-maximum = 2 Hz).

S1.5 $CH_4@C_{60}$

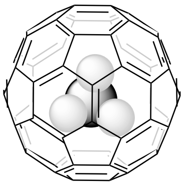

Triisopropyl phosphite (36 μL, 0.147 mmol) was added to a solution of CH_4@**6** (10.0 mg, 0.00920 mmol) in toluene (2 mL) and the resulting mixture was stirred at reflux for 21 h. After cooling to room temperature, the mixture was concentrated *in vacuo* and purified by column chromatography (SiO_2 eluted with toluene).[6] The fractions containing material with $R_f = 0.95$ were collected and evaporated to dryness to afford a black solid which was taken into 1-chloronaphthalene (1 mL) and transferred to a Schlenk flask charged with N-phenyl maleimide (2 mg, 0.0113 mmol) and fitted with a straight condenser. The mixed solution was degassed and placed under an atmosphere of argon before stirring at reflux for 24 h. After cooling to room temperature, the mixture was flushed through a SiO_2 column packed with toluene, collecting a purple band. Purification by preparative HPLC on a Cosmosil™ Buckyprep column (eluted with toluene) gave the title compound $CH_4@C_{60}$, with 100.0 ± 0.3 % filling, as a black solid (7.1 mg, quant. yield). Although this step gave a quantitative measured yield there is likely to be a significant error associated with the very small quantity of material.

^{1}H NMR (700 MHz, 1,2-dichlorobenzene-d_4) $\delta = -5.71$ (s) ppm.

^{13}C{^{1}H} NMR (176 MHz, 1,2-dichlorobenzene-d_4) $\delta = 143.20, -13.63$ ppm.

^{13}C NMR (176 MHz, 1,2-dichlorobenzene-d_4) $\delta = 143.20, -13.63$ (quintet, $^1J_{HC} = 124.25 \pm 0.20$ Hz, endohedral CH_4) ppm.

HRMS-APPI (*m/z*): Calcd. for $[^{12}C_{61}H_4]^{+\bullet}$, 736.0308; found 736.0314. Calcd. for $[^{12}C_{60}{}^{13}CH_4]^{+\bullet}$ isotope, 737.0341; found 737.0349. Calcd. for $[^{12}C_{59}{}^{13}C_2H_4]^{+\bullet}$ isotope, 738.0375; found 738.0380. Calcd. for $[^{12}C_{58}{}^{13}C_3H_4]^{+\bullet}$ isotope, 739.0408; found 739.0406.

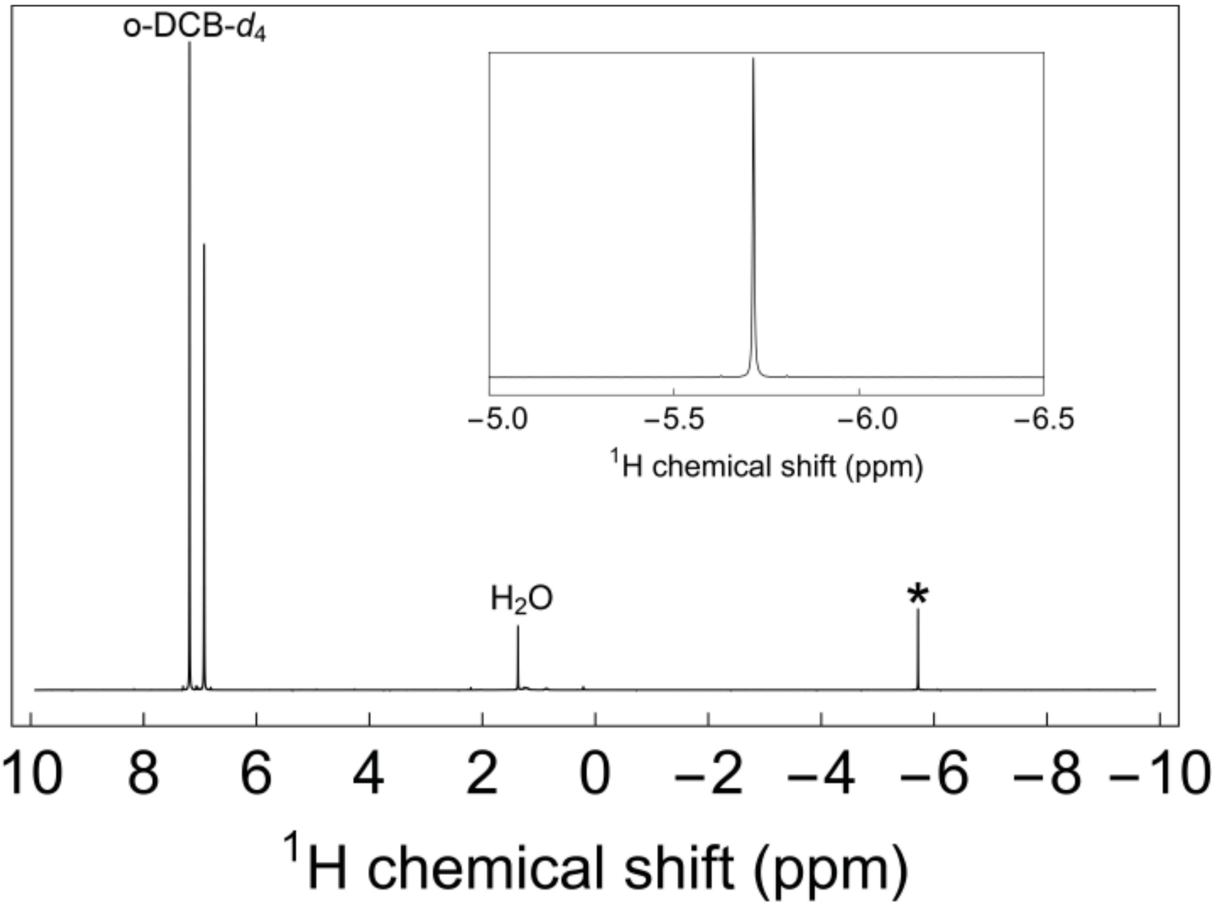

Figure S1.4 Experimental ^{1}H NMR spectrum of $CH_4@C_{60}$ (4.5 mM in degassed 1,2-dichlorobenzene-d_4) acquired at 16.45 T (^{1}H nuclear Larmor frequency = 700 MHz) and 295 K with 1 transient. The peak corresponding to $C\underline{H}_4@C_{60}$ is found at −5.71 ppm, marked with an asterisk and shown in the expansion. The spectrum was processed using Lorentzian line broadening (full-width at half-maximum = 1.75 Hz). A peak from residual H_2O dissolved in 1,2-dichlorobenzene-d_4 is located at 1.37 ppm.[6]

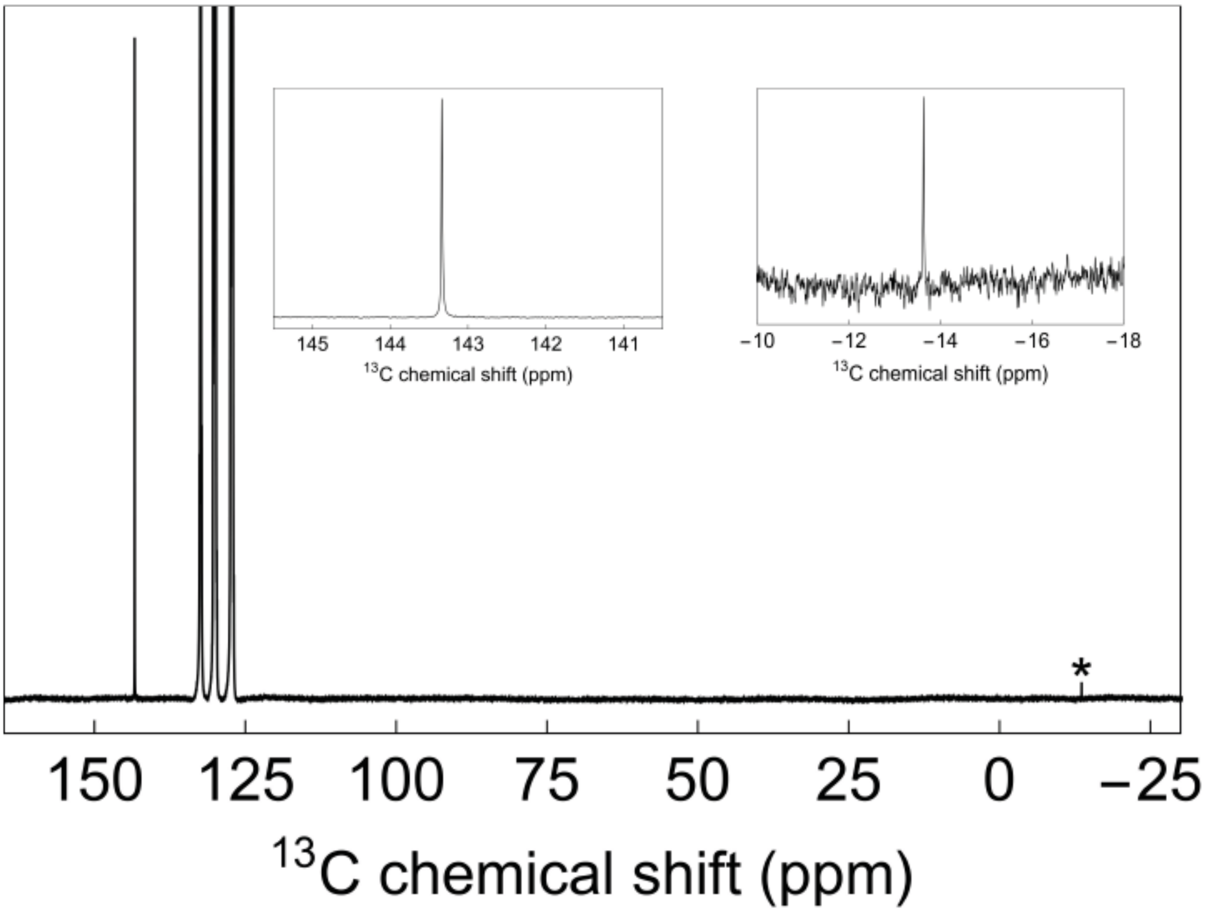

Figure S1.5 Experimental ^{13}C NMR spectrum of $CH_4@C_{60}$ with ^{1}H WALTZ16 decoupling (nutation frequency = 14.2 kHz) of $CH_4@C_{60}$ (4.5 mM in degassed 1,2-dichlorobenzene-d_4) acquired at 16.45 T (^{13}C nuclear Larmor frequency = 176 MHz) and 295 K with 4928 transients and a delay of 10 s between scans. The spectrum was processed using Lorentzian line broadening (full-width at half-maximum = 2 Hz). The three solvent peaks are found around 130 ppm. The ^{13}C $CH_4@\underline{C}_{60}$ peak is found at 143.34 ppm and the $^{13}\underline{C}H_4@C_{60}$ peak at −13.63 ppm (marked with an asterisk); both resonances are shown in the expansions.

S2. ^{13}C NMR simulation using *SpinDynamica*

Simulation of the non-proton-decoupled ^{13}C NMR spectrum of $CH_4@C_{60}$ (Figure 4e of the main paper) was carried out using *SpinDynamica* code for Mathematica programmed by Malcolm H. Levitt, with contributions by Jyrki Rantaharju, Andreas Brinkmann and Soumya Singha Roy, available at: www.spindynamica.soton.ac.uk.[7]

The simulated spectrum was obtained using a chemical shift Hamiltonian = $2\pi\, \Omega\, \hat{I}_Z$ where $\Omega = -13.63$ ppm (at a ^{13}C Larmor frequency of 176 MHz) and a J-coupling Hamiltonian = $2\pi\, J_{HC}\, \hat{I}_{Hz} \cdot \hat{I}_{Cz}$ where J_{HC} = 124.25 ± 0.20 Hz or 124.3 ± 0.2 Hz ($\hat{I}_z = \hat{I}_{Cz} + \hat{I}_{Hz}$ and $\hat{I}_{Hz} = \hat{I}_{1Hz} + \hat{I}_{2Hz} + \hat{I}_{3Hz} + \hat{I}_{4Hz}$).

S3. Measurement of spin-lattice relaxation (T_1)

S3.1 Experimental ^{1}H spin-lattice relaxation curves

Experimental ^{1}H spin-lattice relaxation (T_1) curves for $^{12}C\underline{H}_4@C_{60}$ at 295, 300, 305, 310 and 315 K; are shown in Figure S3.1 below.

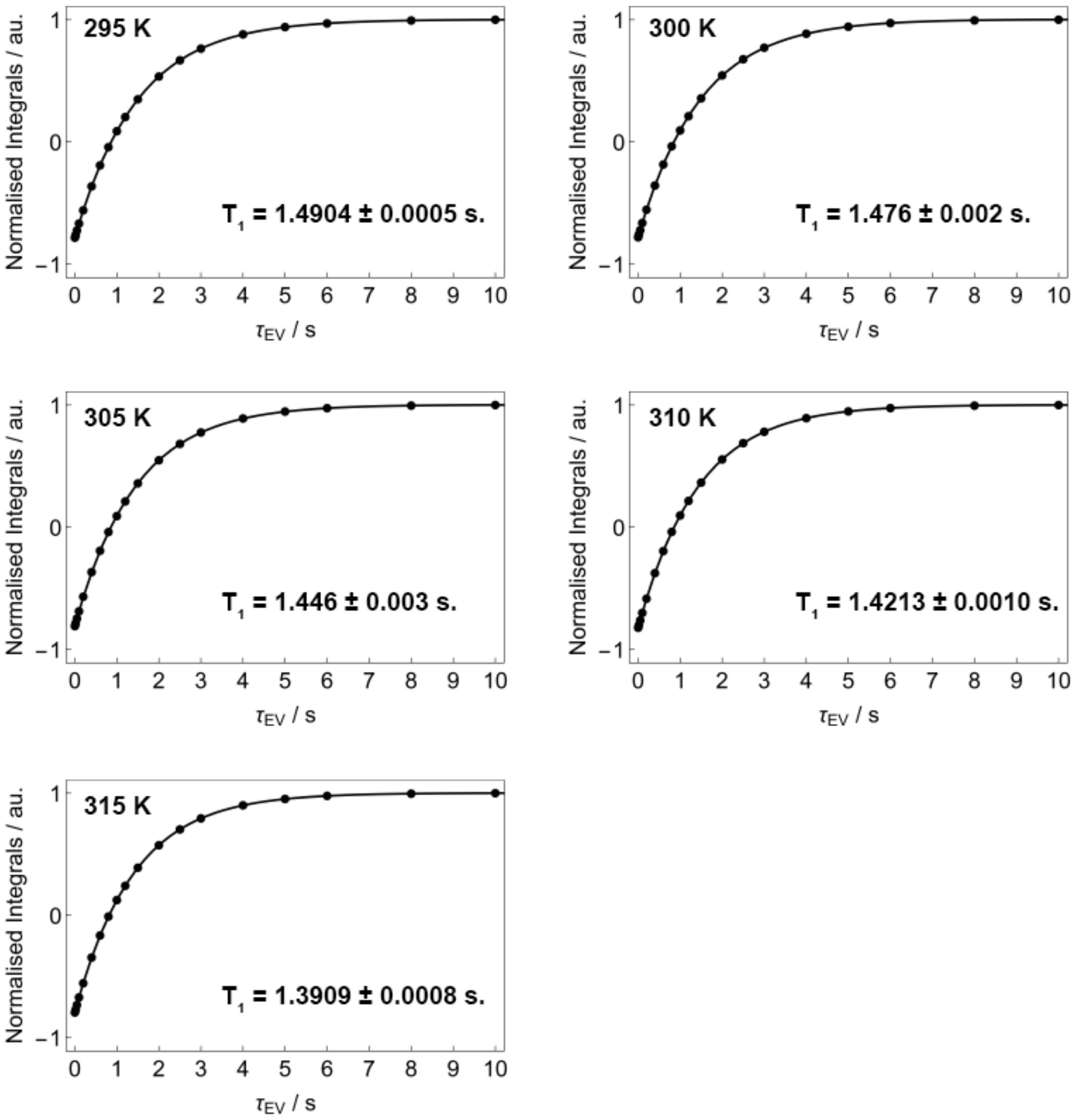

Figure S3.1 Experimental ^{1}H spin-lattice relaxation curves for $^{12}C\underline{H}_4@C_{60}$ as a function of evolution delay, for a 4.5 mM sample of $CH_4@C_{60}$ in degassed 1,2-dichlorobenzene-d_4, acquired at 16.45 T (700 MHz) with 20 transients per data point. ^{1}H longitudinal relaxation times were measured using the inversion-recovery pulse sequence.

S3.2 ^{13}C spin-lattice relaxation measurement using INEPT

In order to obtain ^{13}C T_1 measurements using a 4.5 mM solution of un-labelled $CH_4@C_{60}$ in 1,2-dichlorobenzene-d_4, the ^{1}H magnetisation was transferred to ^{13}C through a refocused INEPT sequence for an IS_4 system,[8] followed by a variable τ_{EV} delay in which the ^{13}C magnetisation decays. The remaining ^{13}C magnetisation was transferred to the protons through a reversed INEPT sequence, generating an antiphase pattern for the ^{13}C satellites. Detection was made through the sensitive proton channel. The pulse sequence used is shown in Figure S3.2, and Figure S3.3 shows the two anti-phase satellite peaks with τ_{EV} = 1ms, $\tau_1 = \frac{1}{4\,J_{HC}}$ and $\tau_2 = \frac{1}{12\,J_{HC}}$, (J_{HC} = 124.3 ± 0.2 Hz). The peak intensities oscillate and are not always equal, however the maximum intensity for both satellites is seen at 1 ms τ_{EV} delay and not 100 μs which is the shortest delay in the sequence.

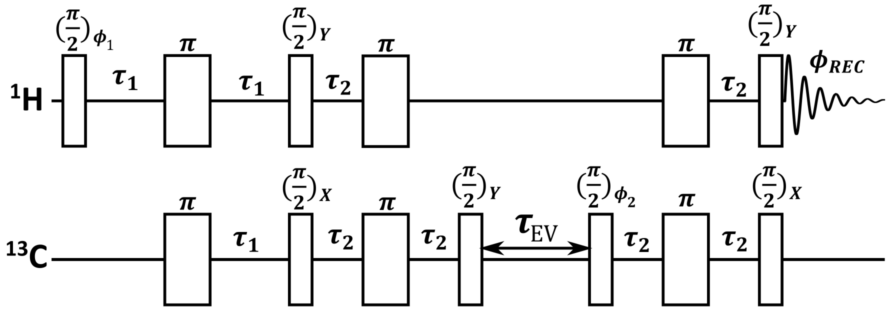

Figure S3.2 Pulse sequence used to monitor the decay of ^{13}C longitudinal spin order in $^{13}\underline{C}H_4@C_{60}$. The decay of ^{13}C longitudinal spin order is tracked by repeating the experiment for different values of the evolution period τ_{EV}. The sequence uses a four-step phase cycle to remove residual proton magnetisation (ϕ_1 = (x,x,x,-x), ϕ_2 = (y,y,-y,-y), ϕ_{REC} = (x,-x,-x,x)). A delay of 8 s was used between successive experiments. The experimental parameters were as follows:[8] $\tau_1 = \frac{1}{4\,J_{HC}}$ = 2 ms and $\tau_2 = \frac{1}{12\,J_{HC}}$ = 0.67 ms , (J_{HC}= 124.3 ± 0.2 Hz).

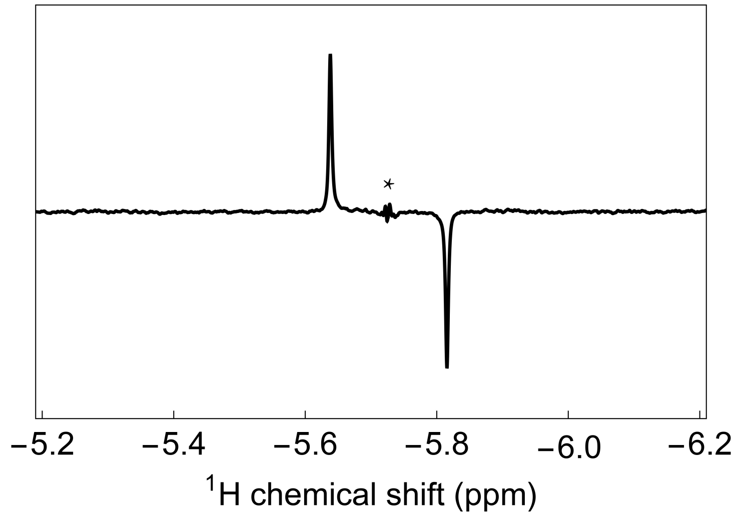

Figure S3.3 Relevant part of the experimental ^{1}H NMR spectrum of $^{13}C\underline{H}_4@C_{60}$ (natural abundance), from a 4.5 mM solution of $CH_4@C_{60}$ in degassed 1,2-dichlorobenzene-d_4; acquired at 16.45 T (^{1}H nuclear Larmor frequency = 700 MHz) and 295 K with 64 transients (with 8 s between transients) after application of the pulse sequence in Figure S3.2 with an evolution period τ_{EV} = 1 ms. The spectrum was processed using Lorentzian line broadening (full-width at half-maximum = 1.5 Hz). The asterisk denotes the $^{12}CH_4@C_{60}$ proton peak removed by the application of the four-step phase cycle. The small residual peak (*) indicates a strong suppression of the $^{12}CH_4@C_{60}$ proton signal attributed to the implementation of the four-step phase cycle.

S4. Mass spectrometry

Figure S4.1 shows the positive ion atmospheric pressure photoionisation (APPI) mass spectrum for a mixture of $CH_4@C_{60}$ and $H_2O@C_{60}$. Ultrahigh resolution (132,000 at *m/z* 738) is required to show the individual species at *m/z* 738.0097 ($[^{12}C_{59}{}^{13}C_2H_4]^{+\bullet}$) and 738.0378 ($[^{12}C_{60}H_2O]^{+\bullet}$). At the same resolution, there is no evidence of $H_2O@C_{60}$ (*m/z* 738.0100) in the APPI mass spectrum of $CH_4@C_{60}$ (Figure 2, main text).

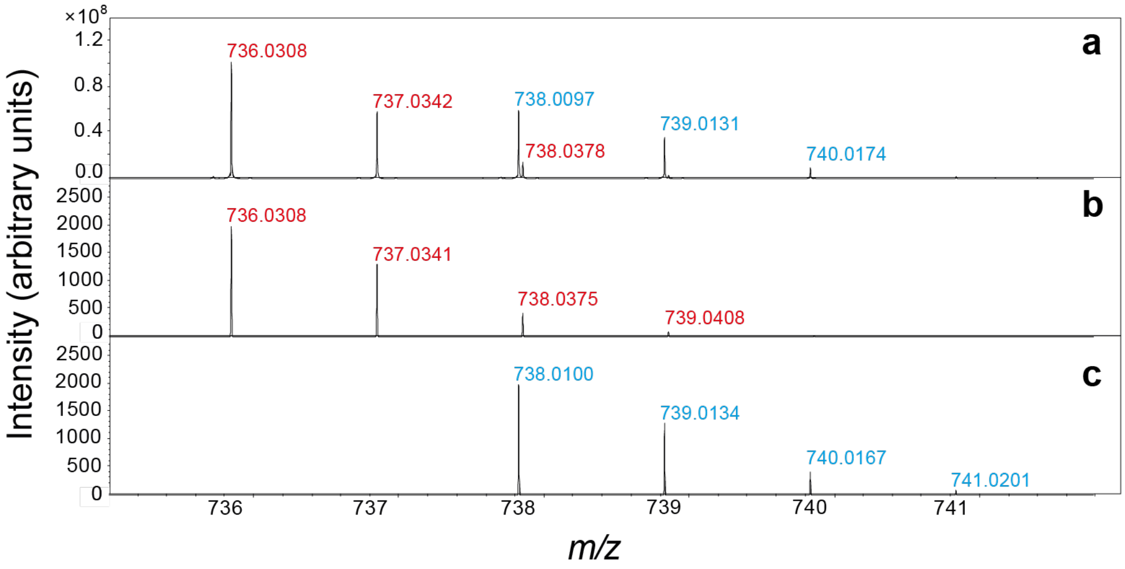

Figure S4.1 Positive ion atmospheric pressure photoionisation (APPI) mass spectra of $CH_4@C_{60}$ and $H_2O@C_{60}$; (a) Experimental data for a mixed sample of $CH_4@C_{60}$ (red labels) and $H_2O@C_{60}$ (blue labels); (b) model isotope pattern for $C_{61}H_4$; (c) model isotope pattern for $C_{60}H_2O$.

S5. Measurement of the relative yields for photochemical closure of CH_4@5 vs H_2O@5

We were unable to attribute the low yielding photochemical ring-contraction of CH_4@**5** to any factor other than the presence of endohedral methane and tested the proposal using partially filled **5** where we would expect a change in filling factor to be a sensitive test of the effect of the methane, *i.e.* if the closure of 'empty' **5** is higher yielding than that of CH_4@**5** we would expect the filling factor to be lower in the product **4**, after closure. Unfortunately, given the very fast entry of H_2O into **5** under the partially aqueous conditions of the reaction, the competition is mostly between CH_4@**5** and H_2O@**5** (rather than between CH_4@**5** and empty **5**) so we are unable to distinguish between an inhibitory effect of the CH_4 and a promoting effect of the H_2O. The precise equilibrium filling of **5** with H_2O under the reaction conditions is unknown, but closure of 'empty' **5** occurs to yield product **4** containing 80 ± 5% endohedral H_2O in ~25% yield.

S5.1 Experimental method

A mixture of CH_4@**5** (>95% filling, 32 mg) and **5** (10 mg) was prepared in order to dilute the filled material. The resulting sample was measured as comprising 83% CH_4@**5** and 17% H_2O@**5** by comparison of integrals in the experimental ^{1}H NMR spectrum, acquired at 400 MHz in $CDCl_3$, with a pulse delay of d_1 = 45 s to ensure full relaxation of the endohedral nuclei. The diluted sample was irradiated for 24 h under the conditions described in S1.4. A mixture of products CH_4@**4** and H_2O@**4** combined with residual starting materials CH_4@**5** and H_2O@**5**, was obtained by column chromatography (SiO_2 eluted with a 90:8:2 mixture of toluene:EtOAc:AcOH). Due to the requirement for long ^{1}H NMR data acquisition time and the instability of bis(hemiketal) **4** upon exposure to air and light in $CDCl_3$, this mixture was directly reduced as follows: after dissolution in toluene (1 mL), triphenylphosphine (45 mg, 0.173 mmol, approx. 20 equiv.) was added and the resulting mixture stirred at reflux for 18 h with exclusion of light. After cooling to room temperature, solvents were removed *in vacuo*. Purification by column chromatography (SiO_2 eluted with a gradient of 1:1 hexane:toluene → toluene) gave a mixture of CH_4@**6** and H_2O@**6** as a shiny brown/black solid (1.4 mg) which is stable in $CDCl_3$ solution. A methane filling of 57% and amplified water filling of 41% was determined by comparison of integrals in the ^{1}H NMR spectrum.

We assume that the % filling is unaffected in conversion of **4** to **6** since no change in % filling for conversion of H_2O@**4** to H_2O@**6** under identical conditions has been observed.[6] The possibility of release of the entrapped methane molecule during the photochemical ring-contraction is discounted by our observation that there was no measured change in % filling when the CH_4@**5** starting material has >95% filling. Estimation of the error in the calculated ratio CH_4@**4**/H_2O@**4** assumes that the measurement of % filling from the experimental ^{1}H NMR spectrum has a standard deviation of ± 2.5%. The samples may contain a few % empty **6**, but as this is the difference between the two measured incorporations and 100%, the uncertainty is large. We take the total of H_2O@**6** and empty **6** to be 43% for the calculation below.

Relative yields CH_4@**4**/(H_2O@**4** + **4**)= ((57÷43)×(17÷83)) = 0.27 ± 0.08.

In accordance with our observations, it would therefore be expected that the procedure which yields 25% of bis(hemiketal) H_2O@**4** from irradiation of sulfoxide **5**, will furnish 4 – 8% of CH_4@**4** from irradiation of CH_4@**5**.

S6. X-Ray structure determination of $CH_4@C_{60}$

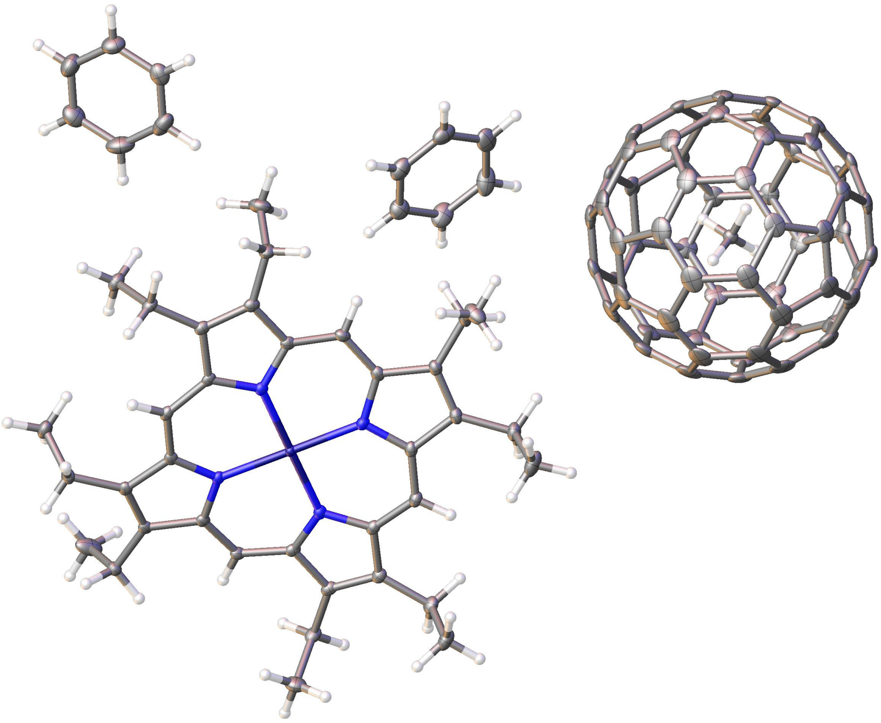

Figure S6.1 Thermal ellipsoid plot of the asymmetric unit, ellipsoids drawn at the 50% probability level with hydrogen atoms depicted as spheres with an arbitrary radius. The hydrogen atoms of methane were attached at calculated positions and the rotation of the rigid group was refined until a local minimum in the shift/error was achieved. As described in the main paper, the visual depiction of hydrogen atoms has no physical significance.

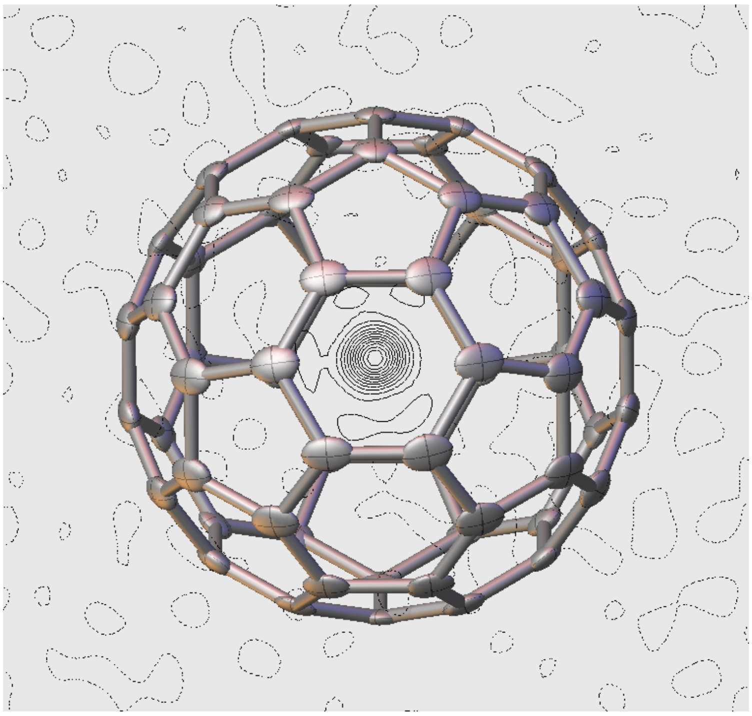

Figure S6.2 Difference electron density map with CH_4 removed from the model. Thermal ellipsoids are drawn at the 50% probability level.

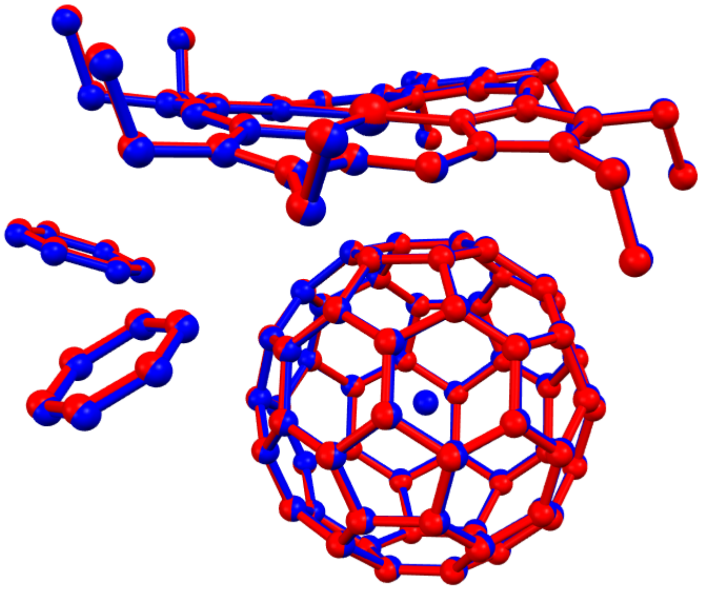

Figure S6.3 Overlay of $CH_4@C_{60}$ reported in this paper (blue) and empty C_{60} (red)[9] structures; the r.m.s difference is 0.00876 (data collection temperatures differ by 8K).

	C…C cross cage	C...C cage to methane
Longest distance (Å)	7.085(3)	3.549(3)
Shortest distance (Å)	7.058(3)	3.525(3)
Difference (Å)	0.027(9)	0.024(9)

Table S6.1 Geometric distortion parameters, in both cases the difference is within 3 sigma of the value and interpreted as insignificant.

S6.1 Experimental method

Single dark orange plate-shaped crystals were recrystallised from a solution of $CH_4@C_{60}$ and nickel(II) octaethylporphyrin (1:1.6 molar ratio of $CH_4@C_{60}$:Ni(II)OEP) in benzene, by slow evaporation.[10] A suitable crystal of 0.10 × 0.10 × 0.02 mm^3 dimensions was selected and mounted on a MITIGEN holder with silicon oil on an Rigaku AFC12 FRE-VHF diffractometer. The crystal was kept at a steady T = 100(2) K during data collection. The structure was solved with the ShelXT structure solution program using the intrinsic phasing methods solution method and by using Olex2[11] as the graphical interface. The model was refined with version 2016/6 of ShelXL[12] using Least Squares minimisation.

S6.2 Crystal data

$C_{109}H_{60}N_4Ni$, M_r = 1484.32, triclinic, *P*-1 (No. 2), a = 14.0857(3) Å, b = 14.3615(3) Å, c = 17.2193(3) Å, α = 87.6060(17)°, β = 75.7006(18)°, γ = 75.677(2)°, V = 3269.79(13) Å^3, T = 100(2) K, Z = 2, Z' = 1, μ(MoKα) = 0.363, 59759 reflections measured, 16567 unique (R_{int} = 0.0498) which were used in all calculations. The final wR_2 was 0.1069 (all data) and R_1 was 0.0509 (I > 2(I)).

CCDC	1858399	Z	2
Formula	$C_{109}H_{60}N_4Ni$	Z'	1
$D_{calc.}$/ g cm^{-3}	1.508	Wavelength/Å	0.71073
μ/mm^{-1}	0.363	Radiation type	MoK$_\alpha$
Formula Weight	1484.32	Θ_{min}/°	1.846
Colour	dark orange	Θ_{max}/°	28.500
Shape	plate	Measured Refl.	59759
Size/mm^3	0.10 × 0.10 × 0.02	Independent Refl.	16567
T/K	100(2)	Reflections with I >2(I)	13160
Crystal System	triclinic	R_{int}	0.0498
Space Group	*P*-1	Parameters	1047
a/Å	14.0857(3)	Restraints	10
b/Å	14.3615(3)	Largest Peak	0.516
c/Å	17.2193(3)	Deepest Hole	-0.574
α/°	87.6060(17)	GooF	1.039
β/°	75.7006(18)	wR_2 (all data)	0.1069
γ/°	75.677(2)	wR_2	0.1001
V/Å^3	3269.79(13)	R_1 (all data)	0.0722
		R_1	0.0509

S6.3 Structure quality indicators

**Reflections:**	d min (Mo) 0.74	I/σ 18.6	Rint 4.98%	complete 100% (IUCr) 100%
**Refinement:**	Shift 0.001	Max Peak 0.5	Min Peak -0.6	GooF 1.039

A dark orange plate-shaped crystal with dimensions 0.10×0.10×0.02 mm^3 was mounted on a MITIGEN holder with silicon oil. X-ray diffraction data were collected using a Rigaku AFC12 FRE-VHF diffractometer equipped with an Oxford Cryosystems low-temperature device, operating at T = 100(2) K.

Data were measured using profile data from ω-scans of 0.5 ° per frame for 15.0 s using MoKα radiation (Rotating-anode X-ray tube, 45.0 kV, 55.0 mA). The total number of runs and images was based on the strategy calculation from the program **CrysAlisPro** (Rigaku, V1.171.39.46, 2018). The maximum resolution achieved was θ = 28.500°.

Cell parameters were retrieved using the **CrysAlisPro** (Rigaku, V1.171.39.46, 2018) software and refined using **CrysAlisPro** (Rigaku, V1.171.39.46, 2018) on 16292 reflections, 27 % of the observed reflections. Data reduction

was performed using the **CrysAlisPro** (Rigaku, V1.171.39.46, 2018) software that corrects for Lorentz polarisation. The final completeness is 100.00 % out to 28.500° in θ.

A multi-scan absorption correction was performed using CrysAlisPro 1.171.39.46 (Rigaku Oxford Diffraction, 2018) using spherical harmonics as implemented in SCALE3 ABSPACK. The absorption coefficient μ of this material is 0.363 mm^{-1} at this wavelength (λ = 0.711Å) and the minimum and maximum transmissions are 0.727 and 1.000, respectively.

The structure was solved in the space group *P*-1 (# 2) by intrinsic phasing methods using the ShelXT structure solution program and refined by Least Squares using version 2016/6 of **ShelXL**.[12] All non-hydrogen atoms were refined anisotropically. Most hydrogen atom positions were calculated geometrically and refined using the riding model, but some hydrogen atoms were refined freely.

_refine_special_details: Hydrogen atoms of the CH_4 were generated from observed electron density peaks and then restrained to have tetrahedral geometry, the molecule will be freely rotating and the orientation represents one of many local minima in the LS refinement.

_exptl_absorpt_process_details: CrysAlisPro 1.171.39.46 (Rigaku Oxford Diffraction, 2018) using spherical harmonics as implemented in SCALE3 ABSPACK.

There is a single molecule in the asymmetric unit, which is represented by the reported sum formula. In other words: Z is 2 and Z' is 1.

S6.4 Generated precession images

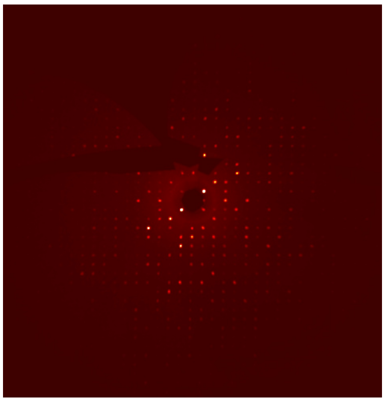

0kl

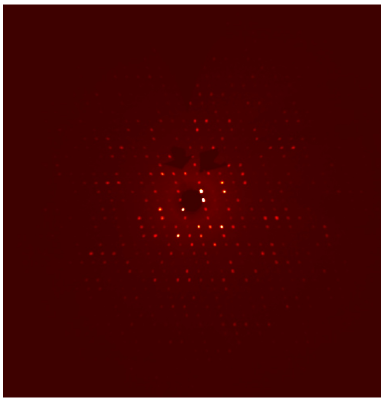

h0l

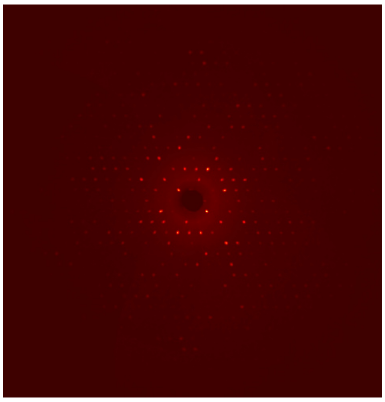

hk0

S6.5 Data plots: diffraction data

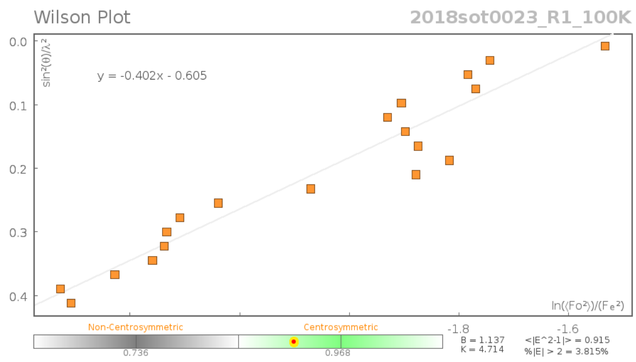

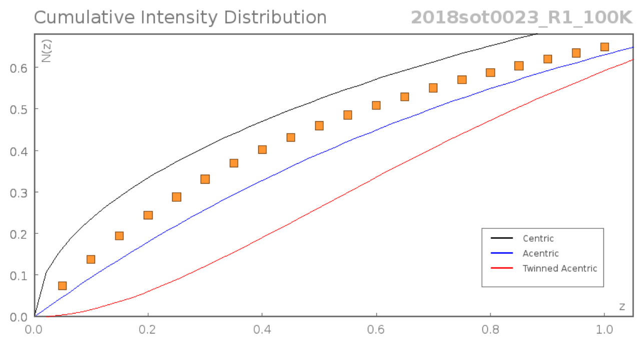

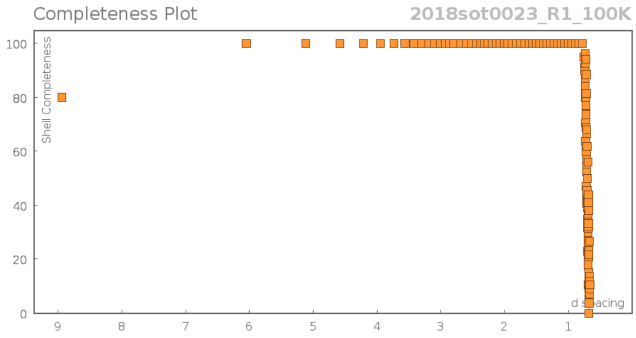

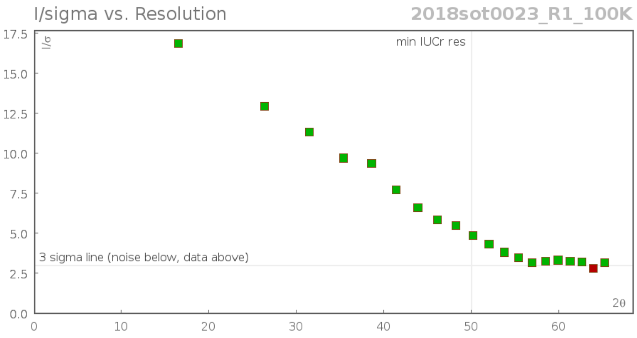

S6.6 Data plots: refinement and data

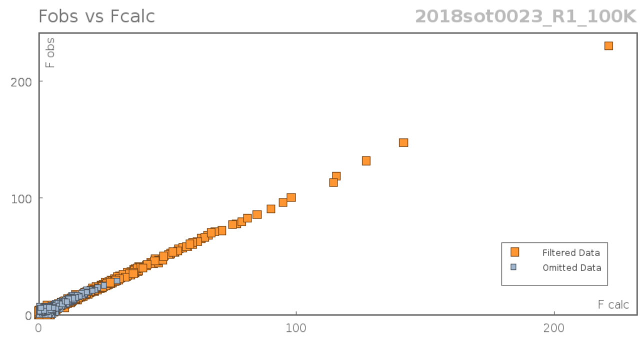

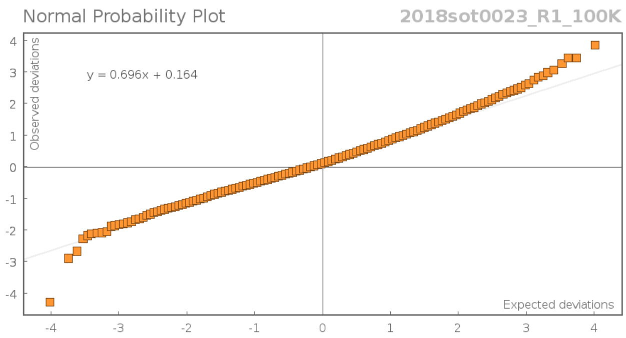

S6.7 Reflection statistics

Total reflections (after filtering)	59759	Unique reflections	16567
Completeness	1.0	Mean I/σ	14.39
hkl_{max} collected	(21, 21, 21)	hkl_{min} collected	(-20, -21, -25)
hkl_{max} used	(18, 19, 23)	hkl_{min} used	(-18, -19, 0)
Lim d_{max} collected	100.0	Lim d_{min} collected	0.74
d_{max} used	11.03	d_{min} used	0.74
Friedel pairs	12146	Friedel pairs merged	1
Inconsistent equivalents	1	R_{int}	0.0498
R_{sigma}	0.0539	Intensity transformed	0
Omitted reflections	0	Omitted by user (OMIT hkl)	0
Multiplicity	(12322, 11578, 5273, 2275, 592, 169, 4)	Maximum multiplicity	9
Removed systematic absences	0	Filtered off (Shel/OMIT)	4640

Table S6.2: Fractional Atomic Coordinates (×10⁴) and Equivalent Isotropic Displacement Parameters (Å²×10³). U_{eq} is defined as 1/3 of the trace of the orthogonalised U_{ij}.

Atom	x	y	z	U_{eq}
Ni1	4655.6(2)	5862.8(2)	8875.1(2)	9.82(6)
N1	4163.1(11)	5251.4(11)	8119.0(9)	12.8(3)
N2	3484.4(11)	6946.5(11)	8996.9(9)	12.5(3)
N3	5157.4(11)	6484.6(11)	9622.6(9)	11.2(3)
N4	5836.8(11)	4783.2(11)	8740.8(9)	11.9(3)
C62	4668.6(14)	4454.8(13)	7631.5(10)	13.9(4)
C63	4055.2(14)	4233.4(13)	7138.8(11)	15.0(4)
C64	3145.7(15)	4878.5(14)	7351.1(11)	16.1(4)
C65	3225.6(14)	5519.7(13)	7943.8(10)	14.0(4)
C66	2493.6(14)	6335.3(13)	8247.6(11)	15.0(4)
C67	2632.8(13)	7021.8(13)	8715.3(10)	13.2(3)
C68	1925.4(14)	7941.8(13)	8927.5(11)	14.9(4)
C69	2369.2(14)	8453.2(13)	9315.3(11)	14.5(4)
C70	3317.6(13)	7822.0(13)	9372.7(10)	12.5(3)
C71	3941.1(13)	8043.1(13)	9807.3(10)	13.1(3)
C72	4785.0(13)	7402.7(13)	9946.1(10)	12.6(3)
C73	5379.8(13)	7600.9(13)	10467.8(10)	12.8(3)
C74	6118.6(14)	6790.0(13)	10466.1(10)	13.3(4)
C75	5974.8(13)	6106.3(13)	9939.5(10)	12.3(3)
C76	6617.8(13)	5213.3(13)	9746.2(10)	12.6(3)
C77	6577.2(13)	4611.8(13)	9161.2(10)	12.2(3)
C78	7351.9(14)	3749.3(13)	8871.3(11)	13.7(4)
C79	7084.4(14)	3395.2(13)	8258.4(11)	14.4(4)
C80	6144.5(13)	4033.1(13)	8187.7(10)	12.8(3)
C81	5609.1(14)	3899.1(13)	7648.8(11)	14.9(4)
C82	4386.1(15)	3438.7(14)	6522.0(12)	19.7(4)
C83	4216.3(19)	2478.6(15)	6846.1(13)	29.2(5)
C84	2224.6(15)	4946.3(15)	7039.9(12)	20.4(4)
C85	1447.6(17)	4490.4(18)	7591.4(15)	32.0(5)
C86	896.2(14)	8223.3(14)	8766.1(12)	17.8(4)
C87	125.4(15)	7806.9(16)	9372.2(13)	24.3(4)
C88	1958.2(14)	9458.2(13)	9656.2(12)	18.0(4)
C89	1402.6(17)	9493.9(15)	10543.4(13)	26.8(5)
C90	5207.9(14)	8534.0(13)	10902.9(11)	15.8(4)
C91	5920.1(19)	9145.2(16)	10493.7(14)	33.1(5)
C92	6965.2(14)	6610.7(14)	10880.0(11)	15.9(4)
C93	7971.6(15)	6709.8(16)	10331.0(12)	21.2(4)
C94	8279.5(14)	3386.1(13)	9179.4(11)	15.6(4)
C95	9095.0(15)	3933.2(15)	8854.4(12)	20.1(4)
C96	7642.0(15)	2519.8(14)	7743.0(12)	19.6(4)
C97	8446.6(17)	2713.3(17)	7027.3(13)	29.0(5)
C1	6527.1(18)	10244.4(15)	5699.0(14)	28.6(5)
C2	7192.2(19)	10491.0(14)	6138.5(14)	27.4(5)
C3	8234.0(18)	10203.0(15)	5840.0(13)	25.5(5)
C4	8653.1(18)	9654.5(16)	5093.3(13)	28.7(5)
C5	8014(2)	9417.3(18)	4673.5(13)	32.2(5)
C6	6932.3(19)	9721.5(17)	4982.4(13)	30.5(5)
C7	6502.1(19)	8950.7(17)	4813.8(13)	31.0(5)
C8	5689.0(18)	8743.6(17)	5368.9(14)	29.3(5)
C9	5265.6(16)	9290.3(16)	6118.0(14)	27.9(5)
C10	5675.8(17)	10027.2(15)	6276.6(14)	28.5(5)
C11	5814.7(16)	10137.1(15)	7073.3(14)	26.0(5)
C12	6750.5(17)	10428.0(14)	6987.3(13)	24.9(5)
C13	7370.9(17)	10079.1(14)	7503.4(12)	23.9(4)
C14	8452.5(17)	9780.7(15)	7193.1(13)	23.4(4)
C15	8878.7(17)	9839.0(15)	6377.9(13)	25.0(5)
C16	9699.4(16)	9063.2(17)	5966.1(13)	27.3(5)
C17	9559.1(18)	8950.7(18)	5170.2(13)	30.5(5)
C18	9789.3(17)	8036.7(19)	4831.4(13)	33.1(5)

Atom	x	y	z	U_{eq}
C19	9126.2(19)	7790.2(19)	4391.7(12)	33.1(6)
C20	8264(2)	8461.9(19)	4315.5(12)	33.2(6)
C21	7326(2)	8178.5(18)	4402.0(12)	32.3(5)
C22	7302.0(19)	7228.1(18)	4562.1(12)	31.0(5)
C23	6440.7(18)	7015.1(17)	5147.3(13)	27.8(5)
C24	5659.1(17)	7753.2(17)	5537.8(13)	26.9(5)
C25	5219.5(15)	7689.7(16)	6388.0(13)	23.9(4)
C26	4974.7(15)	8639.2(16)	6746.7(14)	25.9(5)
C27	5109.4(15)	8744.7(16)	7507.4(13)	23.6(4)
C28	5538.6(16)	9513.4(15)	7672.4(13)	25.2(5)
C29	6183.2(16)	9148.9(15)	8212.4(11)	22.5(4)
C30	7077.3(16)	9429.7(15)	8133.2(11)	22.1(4)
C31	7978.5(16)	8727.7(16)	8213.3(11)	22.4(4)
C32	8833.6(16)	8940.4(16)	7632.0(12)	23.1(4)
C33	9612.5(16)	8202.7(16)	7238.2(13)	25.4(5)
C34	10055.5(16)	8263.6(17)	6389.1(14)	27.9(5)
C35	10296.0(15)	7312.8(18)	6033.5(14)	30.7(5)
C36	10167.5(16)	7202.9(18)	5272.0(14)	32.8(5)
C37	9736.4(17)	6436.2(18)	5105.8(14)	33.7(5)
C38	9103.4(19)	6793.7(18)	4558.5(13)	34.0(6)
C39	8201.3(19)	6520.7(17)	4643.6(13)	31.7(5)
C40	7909.3(18)	5868.0(16)	5272.8(13)	29.5(5)
C41	6816.5(18)	6178.6(15)	5582.3(13)	26.6(5)
C42	6398.3(17)	6115.4(15)	6396.3(13)	24.3(4)
C43	5575.9(15)	6892.0(15)	6809.6(12)	22.3(4)
C44	5716.2(15)	7000.2(15)	7607.1(12)	22.1(4)
C45	5487.0(15)	7911.3(16)	7945.8(12)	21.9(4)
C46	6149.6(16)	8161.6(16)	8381.9(11)	21.8(4)
C47	7013.2(17)	7490.2(16)	8458.2(11)	22.2(4)
C48	7947.3(16)	7777.9(16)	8370.1(11)	23.3(4)
C49	8767.3(16)	7005.6(16)	7959.5(13)	24.6(4)
C50	9583.4(16)	7211.6(17)	7408.1(14)	27.2(5)
C51	10006.3(16)	6661.1(17)	6663.5(15)	30.1(5)
C52	9594.7(17)	5929.7(16)	6500.9(15)	30.7(5)
C53	9460.4(17)	5815.6(16)	5709.8(15)	32.7(5)
C54	8522.1(18)	5526.5(15)	5794.0(14)	30.3(5)
C55	8079.9(18)	5459.0(14)	6640.2(14)	27.7(5)
C56	7042.4(17)	5750.0(14)	6937.0(13)	23.7(4)
C57	6625.7(16)	6299.1(15)	7681.8(12)	22.0(4)
C58	7260.1(17)	6534.8(15)	8101.8(12)	22.9(4)
C59	8339.6(17)	6238.8(15)	7793.9(13)	26.0(5)
C60	8744.5(17)	5709.6(15)	7081.0(14)	27.5(5)
C61	7639.2(14)	7976.2(14)	6384.4(11)	14.4(4)
C98	3126.0(17)	6325.7(16)	4660.1(14)	26.8(5)
C99	2631.7(17)	7283.2(16)	4795.5(14)	28.0(5)
C100	2658.5(16)	7761.9(17)	5466.7(15)	30.2(5)
C101	3187.8(18)	7282.7(18)	6003.8(14)	31.9(5)
C102	3711.4(19)	6330.9(18)	5854.4(15)	34.2(6)
C103	3668.0(19)	5851.5(16)	5191.8(15)	32.7(5)
C104	2336.5(18)	9894.2(16)	7317.4(14)	30.6(5)
C105	1347.4(18)	9983.1(16)	7304.2(12)	27.0(5)
C106	624.7(17)	10789.5(19)	7625.3(14)	32.7(5)
C107	890.4(19)	11512.6(18)	7959.6(15)	37.7(6)
C108	1881(2)	11426.5(17)	7974.6(14)	33.8(5)
C109	2606.4(18)	10616.7(18)	7650.4(15)	34.2(5)

Table S6.3: Anisotropic Displacement Parameters (×10[4]). The anisotropic displacement factor exponent takes the form: $-2\pi^2[h^2a^{*2} \times U_{11} + \ldots + 2hka^* \times b^* \times U_{12}]$

Atom	U_{11}	U_{22}	U_{33}	U_{23}	U_{13}	U_{12}
Ni1	8.59(11)	10.66(11)	10.55(11)	-0.22(8)	-2.07(8)	-3.16(8)
N1	10.7(7)	13.3(8)	14.6(7)	0.7(6)	-2.5(6)	-3.9(6)
N2	12.1(7)	13.9(8)	12.7(7)	1.6(6)	-2.7(6)	-5.7(6)
N3	9.7(7)	13.2(7)	11.5(7)	2.0(6)	-2.0(6)	-4.7(6)
N4	11.3(7)	12.7(7)	12.5(7)	1.3(6)	-2.1(6)	-5.1(6)
C62	15.9(9)	15.4(9)	11.5(8)	1.1(7)	-2.4(7)	-7.2(7)
C63	17.1(9)	17.4(9)	12.5(8)	0.9(7)	-4.1(7)	-7.3(8)
C64	18.9(9)	17.1(9)	15.7(9)	0.8(7)	-7.1(7)	-7.7(8)
C65	14.6(9)	16.9(9)	12.9(8)	3.1(7)	-4.7(7)	-7.4(7)
C66	13.3(9)	17.6(9)	16.0(9)	3.0(7)	-5.5(7)	-5.4(7)
C67	11.7(8)	15.4(9)	12.6(8)	3.9(7)	-2.8(7)	-4.4(7)
C68	14.0(9)	16.2(9)	14.6(9)	3.6(7)	-3.7(7)	-4.1(7)
C69	12.1(9)	13.6(9)	16.2(9)	3.7(7)	-1.1(7)	-3.1(7)
C70	10.8(8)	11.8(9)	13.9(8)	2.5(6)	0.1(7)	-4.4(7)
C71	12.5(8)	10.8(8)	15.2(9)	-1.2(6)	-0.1(7)	-4.7(7)
C72	11.2(8)	14.7(9)	11.3(8)	0.5(6)	0.5(6)	-5.6(7)
C73	11.7(8)	14.9(9)	12.1(8)	-0.6(7)	0.2(7)	-6.5(7)
C74	12.9(9)	17.5(9)	9.7(8)	1.6(7)	-0.8(7)	-6.1(7)
C75	11.8(8)	15.5(9)	10.1(8)	2.2(6)	-0.5(6)	-7.0(7)
C76	10.6(8)	15.6(9)	12.0(8)	4.7(6)	-3.0(6)	-4.4(7)
C77	9.3(8)	13.5(9)	13.5(8)	3.7(6)	-0.9(6)	-4.9(7)
C78	11.7(9)	13.0(9)	15.7(9)	3.0(7)	-1.2(7)	-4.3(7)
C79	12.9(9)	13.5(9)	15.8(9)	0.9(7)	-0.1(7)	-5.2(7)
C80	12.2(8)	12.1(9)	13.7(8)	1.6(6)	-0.2(7)	-5.4(7)
C81	15.8(9)	13.6(9)	14.1(9)	-2.7(7)	0.3(7)	-4.9(7)
C82	19.0(10)	25.0(11)	17.9(9)	-5.4(8)	-6.9(8)	-7.0(8)
C83	44.6(14)	18.4(11)	24.3(11)	-2.6(8)	-13.8(10)	-1.3(10)
C84	18.9(10)	22.9(10)	23.4(10)	0.6(8)	-10.0(8)	-7.5(8)
C85	22.1(11)	37.5(13)	44.5(14)	12.3(11)	-16.7(10)	-15.1(10)
C86	15.9(9)	17.3(10)	21.2(10)	3.3(7)	-8.3(8)	-2.2(8)
C87	15.5(10)	29.5(12)	30.7(11)	6.4(9)	-9.1(8)	-7.8(9)
C88	14.9(9)	11.9(9)	27.3(10)	0.6(7)	-6.6(8)	-2.1(7)
C89	28.2(12)	20.5(11)	26.7(11)	-6.8(8)	-2.0(9)	-0.2(9)
C90	16.2(9)	16.9(9)	14.4(9)	-3.2(7)	-3.1(7)	-4.3(7)
C91	38.7(13)	21.2(11)	35.4(13)	-10.4(9)	8.7(10)	-16.2(10)
C92	17.0(9)	19.1(10)	13.2(9)	0.9(7)	-5.4(7)	-5.7(8)
C93	14.7(9)	28.6(11)	22.3(10)	2.3(8)	-6.3(8)	-7.6(8)
C94	11.9(9)	14.4(9)	19.4(9)	2.4(7)	-3.7(7)	-1.8(7)
C95	13.8(9)	22.1(10)	25.3(10)	3.6(8)	-5.9(8)	-5.5(8)
C96	17.3(10)	15.8(9)	24.0(10)	-5.0(7)	-5.0(8)	-0.3(8)
C97	28.5(12)	29.9(12)	21.2(10)	-7.0(9)	1.8(9)	-0.3(10)
C1	35.9(13)	18.0(11)	35.7(12)	14.5(9)	-18.6(10)	-6.0(9)
C2	41.6(13)	10.3(9)	33.5(12)	8.0(8)	-13.6(10)	-9.1(9)
C3	38.9(13)	16.3(10)	27.5(11)	11.5(8)	-8.5(9)	-19.9(9)
C4	40.0(13)	30.1(12)	21.3(11)	13.1(9)	-3.4(9)	-24.5(11)
C5	48.7(15)	36.9(13)	16.9(10)	16.9(9)	-8.5(10)	-23.7(12)
C6	44.0(14)	31.0(12)	24.0(11)	18.9(9)	-18.7(10)	-15.4(11)
C7	46.6(14)	35.6(13)	21.7(11)	13.6(9)	-23.3(10)	-17.3(11)
C8	29.9(12)	36.2(13)	30.8(12)	9.2(9)	-23.7(10)	-10.0(10)
C9	19.2(10)	27.8(12)	37.8(12)	6.4(9)	-16.8(9)	0.7(9)
C10	27.1(12)	17.7(10)	39.5(13)	5.9(9)	-16.7(10)	4.3(9)
C11	22.0(11)	13.6(10)	36.2(12)	-4.3(8)	-4.3(9)	5.3(8)
C12	33.9(12)	8.7(9)	30.9(11)	-3.0(8)	-7.2(9)	-3.3(8)
C13	34.4(12)	15.4(10)	24.1(10)	-7.8(8)	-6.6(9)	-9.0(9)
C14	30.6(11)	20.4(10)	26.5(11)	-1.8(8)	-10.0(9)	-16.3(9)
C15	31.6(12)	22.8(11)	28.8(11)	4.7(8)	-8.0(9)	-21.6(9)
C16	21.0(11)	36.3(13)	30.1(11)	3.1(9)	-1.3(9)	-22.4(10)
C17	30.0(12)	39.7(13)	24.7(11)	7.1(9)	4.5(9)	-26.3(11)
C18	25.5(12)	48.0(15)	21.0(11)	-3.6(10)	13.8(9)	-19.3(11)

Atom	U_{11}	U_{22}	U_{33}	U_{23}	U_{13}	U_{12}
C19	35.9(13)	49.8(15)	11.0(10)	-4.0(9)	10.6(9)	-21.4(12)
C20	50.7(15)	47.0(15)	7.1(9)	6.0(9)	-1.0(9)	-28.3(13)
C21	48.4(15)	47.5(15)	10.3(9)	4.6(9)	-12.6(9)	-24.1(12)
C22	45.6(14)	44.0(14)	11.1(9)	-5.6(9)	-7.5(9)	-23.9(12)
C23	37.6(13)	36.4(13)	19.6(10)	-3.4(9)	-12.3(9)	-22.1(11)
C24	27.3(11)	38.3(13)	26.2(11)	4.3(9)	-17.8(9)	-17.5(10)
C25	15.3(10)	34.3(12)	28.0(11)	0.9(9)	-9.4(8)	-13.1(9)
C26	9.9(9)	32.0(12)	35.8(12)	1.1(9)	-7.3(8)	-3.8(9)
C27	11.1(9)	27.7(11)	26.6(11)	-4.9(8)	3.1(8)	-1.4(8)
C28	18.1(10)	23.5(11)	25.9(11)	-9.8(8)	2.0(8)	4.0(8)
C29	24.0(11)	24.1(11)	14.4(9)	-9.4(8)	3.4(8)	-2.8(9)
C30	30.5(11)	22.8(10)	13.7(9)	-8.4(7)	-3.0(8)	-8.7(9)
C31	29.6(11)	30.8(11)	12.8(9)	-1.1(8)	-9.7(8)	-14.0(9)
C32	26.9(11)	29.8(11)	21.9(10)	0.5(8)	-14.1(9)	-15.9(9)
C33	18.4(10)	34.8(12)	29.9(11)	3.7(9)	-12.2(9)	-13.7(9)
C34	13.8(10)	38.0(13)	35.7(12)	2.4(10)	-5.8(9)	-13.6(9)
C35	8.8(9)	38.9(13)	39.8(13)	-1.7(10)	0.8(9)	-3.8(9)
C36	14.9(10)	38.4(14)	35.3(13)	-8.4(10)	13.7(9)	-6.6(10)
C37	21.8(11)	34.7(13)	32.9(12)	-16.0(10)	11.4(9)	-0.9(10)
C38	35.5(13)	40.2(14)	18.9(11)	-16.9(9)	14.0(9)	-13.3(11)
C39	41.4(14)	34.9(13)	17.3(10)	-15.3(9)	2.8(9)	-13.7(11)
C40	39.6(13)	20.7(11)	26.6(11)	-14.5(9)	2.2(10)	-11.8(10)
C41	37.9(13)	23.1(11)	25.9(11)	-7.2(8)	-7.6(9)	-19.1(10)
C42	30.2(12)	22.0(11)	27.6(11)	-1.0(8)	-6.9(9)	-19.2(9)
C43	18.8(10)	28.1(11)	25.3(10)	0.5(8)	-3.8(8)	-17.0(9)
C44	19.5(10)	29.2(11)	19.4(10)	5.4(8)	1.6(8)	-16.6(9)
C45	13.7(9)	31.8(12)	17.5(10)	-0.3(8)	5.7(7)	-10.2(8)
C46	22.6(10)	32.2(12)	8.6(9)	-2.6(8)	4.6(7)	-10.9(9)
C47	30.3(11)	29.4(11)	9.2(9)	6.7(7)	-3.3(8)	-13.9(9)
C48	29.5(11)	33.3(12)	13.0(9)	5.4(8)	-12.0(8)	-12.2(9)
C49	26.0(11)	28.5(11)	24.6(11)	10.9(8)	-17.2(9)	-7.4(9)
C50	16.9(10)	31.9(12)	36.2(12)	5.5(9)	-17.0(9)	-2.5(9)
C51	11.9(10)	28.7(12)	45.3(14)	2.5(10)	-6.9(9)	2.6(9)
C52	19.1(11)	18.6(11)	47.6(14)	-1.3(9)	-7.1(10)	7.0(9)
C53	24.3(12)	20.3(11)	42.5(14)	-12.3(10)	4.4(10)	4.4(9)
C54	32.6(12)	13.4(10)	38.5(13)	-12.0(9)	1.8(10)	-2.3(9)
C55	34.2(13)	8.0(9)	38.0(12)	0.8(8)	-5.8(10)	-2.8(9)
C56	31.1(12)	12.5(9)	29.6(11)	4.4(8)	-5.0(9)	-12.5(9)
C57	26.7(11)	20.4(10)	21.0(10)	10.0(8)	-2.2(8)	-14.8(9)
C58	30.0(11)	23.1(11)	18.1(10)	12.8(8)	-7.2(8)	-11.5(9)
C59	29.2(12)	20.2(11)	30.3(11)	13.5(8)	-14.8(9)	-4.0(9)
C60	26.7(11)	12.6(10)	40.6(13)	7.3(9)	-10.4(10)	1.1(9)
C61	15.9(9)	14.8(9)	12.4(8)	1.6(7)	-1.9(7)	-5.5(7)
C98	26.6(11)	25.2(11)	30.8(12)	3.7(9)	-6.1(9)	-11.7(9)
C99	21.5(11)	28.4(12)	36.2(12)	10.7(9)	-8.1(9)	-10.6(9)
C100	19.0(11)	24.3(12)	46.1(14)	0.1(10)	-2.6(10)	-8.7(9)
C101	30.0(12)	38.3(14)	33.7(12)	1.7(10)	-5.0(10)	-23.0(11)
C102	40.4(14)	33.9(13)	41.6(14)	19.5(10)	-22.4(11)	-24.2(11)
C103	33.8(13)	19.4(11)	48.7(15)	8.1(10)	-15.0(11)	-10.0(10)
C104	26.3(12)	23.0(11)	36.9(13)	-2.2(9)	-2.9(10)	-0.1(9)
C105	35.2(13)	29.7(12)	20.2(10)	5.5(8)	-6.6(9)	-16.1(10)
C106	18.4(11)	46.4(15)	32.5(12)	10.2(10)	-5.3(9)	-8.9(10)
C107	32.0(13)	28.7(13)	39.4(14)	-1.5(10)	4.5(11)	3.4(10)
C108	42.1(14)	27.3(12)	34.2(13)	-2.4(10)	-7.3(11)	-14.3(11)
C109	23.7(12)	37.4(14)	44.0(14)	1.1(11)	-12.1(10)	-8.4(10)

Table S6.4: Bond Lengths in Å.

Atom	Atom	Length/Å
Ni1	N1	1.9469(15)
Ni1	N2	1.9426(15)
Ni1	N3	1.9523(14)
Ni1	N4	1.9466(15)
N1	C62	1.379(2)
N1	C65	1.385(2)
N2	C67	1.380(2)
N2	C70	1.379(2)
N3	C72	1.378(2)
N3	C75	1.377(2)
N4	C77	1.380(2)
N4	C80	1.381(2)
C62	C63	1.445(2)
C62	C81	1.374(3)
C63	C64	1.358(3)
C63	C82	1.497(3)
C64	C65	1.445(2)
C64	C84	1.502(3)
C65	C66	1.377(3)
C66	C67	1.378(3)
C67	C68	1.443(3)
C68	C69	1.360(3)
C68	C86	1.497(3)
C69	C70	1.440(3)
C69	C88	1.501(3)
C70	C71	1.384(2)
C71	C72	1.377(3)
C72	C73	1.450(2)
C73	C74	1.356(3)
C73	C90	1.500(2)
C74	C75	1.446(2)
C74	C92	1.500(2)
C75	C76	1.374(3)
C76	C77	1.375(2)
C77	C78	1.445(3)
C78	C79	1.360(3)
C78	C94	1.498(2)
C79	C80	1.441(3)
C79	C96	1.498(2)
C80	C81	1.378(3)
C82	C83	1.517(3)
C84	C85	1.522(3)
C86	C87	1.528(3)
C88	C89	1.531(3)
C90	C91	1.519(3)
C92	C93	1.533(3)
C94	C95	1.530(3)
C96	C97	1.522(3)
C1	C2	1.453(3)
C1	C6	1.389(3)
C1	C10	1.447(3)
C2	C3	1.391(3)
C2	C12	1.449(3)
C3	C4	1.450(3)
C3	C15	1.448(3)
C4	C5	1.394(3)
C4	C17	1.450(4)
C5	C6	1.443(4)
C5	C20	1.453(3)
C6	C7	1.457(3)

Atom	Atom	Length/Å
C7	C8	1.385(3)
C7	C21	1.450(4)
C8	C9	1.452(3)
C8	C24	1.449(3)
C9	C10	1.390(3)
C9	C26	1.449(3)
C10	C11	1.453(3)
C11	C12	1.451(3)
C11	C28	1.384(3)
C12	C13	1.391(3)
C13	C14	1.444(3)
C13	C30	1.450(3)
C14	C15	1.392(3)
C14	C32	1.456(3)
C15	C16	1.451(3)
C16	C17	1.453(3)
C16	C34	1.388(3)
C17	C18	1.389(3)
C18	C19	1.451(3)
C18	C36	1.450(4)
C19	C20	1.382(4)
C19	C38	1.454(4)
C20	C21	1.448(3)
C21	C22	1.388(3)
C22	C23	1.461(3)
C22	C39	1.448(4)
C23	C24	1.380(3)
C23	C41	1.446(3)
C24	C25	1.451(3)
C25	C26	1.447(3)
C25	C43	1.385(3)
C26	C27	1.389(3)
C27	C28	1.451(3)
C27	C45	1.447(3)
C28	C29	1.451(3)
C29	C30	1.389(3)
C29	C46	1.446(3)
C30	C31	1.445(3)
C31	C32	1.452(3)
C31	C48	1.389(3)
C32	C33	1.380(3)
C33	C34	1.449(3)
C33	C50	1.450(3)
C34	C35	1.447(3)
C35	C36	1.388(3)
C35	C51	1.451(3)
C36	C37	1.451(3)
C37	C38	1.447(4)
C37	C53	1.386(4)
C38	C39	1.393(3)
C39	C40	1.452(3)
C40	C41	1.459(3)
C40	C54	1.388(3)
C41	C42	1.388(3)
C42	C43	1.453(3)
C42	C56	1.451(3)
C43	C44	1.455(3)
C44	C45	1.385(3)
C44	C57	1.451(3)
C45	C46	1.449(3)

Atom	Atom	Length/Å	Atom	Atom	Length/Å
C46	C47	1.384(3)	C57	C58	1.388(3)
C47	C48	1.446(3)	C58	C59	1.440(3)
C47	C58	1.452(3)	C59	C60	1.388(3)
C48	C49	1.447(3)	C98	C99	1.380(3)
C49	C50	1.384(3)	C98	C103	1.386(3)
C49	C59	1.448(3)	C99	C100	1.382(3)
C50	C51	1.448(3)	C100	C101	1.385(3)
C51	C52	1.387(3)	C101	C102	1.384(4)
C52	C53	1.442(4)	C102	C103	1.378(3)
C52	C60	1.450(3)	C104	C105	1.373(3)
C53	C54	1.453(3)	C104	C109	1.379(3)
C54	C55	1.446(3)	C105	C106	1.369(3)
C55	C56	1.385(3)	C106	C107	1.378(4)
C55	C60	1.457(3)	C107	C108	1.377(4)
C56	C57	1.447(3)	C108	C109	1.376(3)

Table S6.5: Bond Angles in °.

Atom	Atom	Atom	Angle/°	Atom	Atom	Atom	Angle/°
N1	Ni1	N3	179.30(7)	C71	C70	C69	123.80(17)
N2	Ni1	N1	90.03(6)	C72	C71	C70	124.16(17)
N2	Ni1	N3	89.92(6)	N3	C72	C73	111.36(15)
N2	Ni1	N4	179.30(7)	C71	C72	N3	124.38(16)
N4	Ni1	N1	89.84(6)	C71	C72	C73	124.24(16)
N4	Ni1	N3	90.20(6)	C72	C73	C90	125.83(16)
C62	N1	Ni1	127.99(12)	C74	C73	C72	106.44(15)
C62	N1	C65	104.08(14)	C74	C73	C90	127.72(16)
C65	N1	Ni1	127.93(12)	C73	C74	C75	106.36(15)
C67	N2	Ni1	127.83(12)	C73	C74	C92	128.89(16)
C70	N2	Ni1	128.12(12)	C75	C74	C92	124.68(16)
C70	N2	C67	104.04(14)	N3	C75	C74	111.64(15)
C72	N3	Ni1	128.11(12)	C76	C75	N3	124.70(16)
C75	N3	Ni1	127.69(12)	C76	C75	C74	123.54(16)
C75	N3	C72	104.20(14)	C75	C76	C77	124.18(17)
C77	N4	Ni1	127.68(12)	N4	C77	C78	111.48(15)
C77	N4	C80	104.19(14)	C76	C77	N4	124.75(16)
C80	N4	Ni1	128.09(12)	C76	C77	C78	123.64(16)
N1	C62	C63	111.51(16)	C77	C78	C94	124.67(16)
C81	C62	N1	124.49(16)	C79	C78	C77	106.28(16)
C81	C62	C63	123.85(17)	C79	C78	C94	128.96(17)
C62	C63	C82	125.37(17)	C78	C79	C80	106.64(16)
C64	C63	C62	106.52(16)	C78	C79	C96	127.84(17)
C64	C63	C82	128.12(17)	C80	C79	C96	125.52(16)
C63	C64	C65	106.49(16)	N4	C80	C79	111.41(15)
C63	C64	C84	128.21(17)	C81	C80	N4	124.29(17)
C65	C64	C84	125.30(17)	C81	C80	C79	124.29(17)
N1	C65	C64	111.33(16)	C62	C81	C80	124.26(17)
C66	C65	N1	124.27(16)	C63	C82	C83	114.43(17)
C66	C65	C64	124.21(17)	C64	C84	C85	112.91(17)
C65	C66	C67	123.97(17)	C68	C86	C87	112.44(16)
N2	C67	C68	111.45(15)	C69	C88	C89	113.01(16)
C66	C67	N2	124.79(17)	C73	C90	C91	113.29(16)
C66	C67	C68	123.65(17)	C74	C92	C93	113.51(15)
C67	C68	C86	124.77(16)	C78	C94	C95	113.11(16)
C69	C68	C67	106.43(16)	C79	C96	C97	113.32(17)
C69	C68	C86	128.76(17)	C6	C1	C2	120.0(2)
C68	C69	C70	106.34(16)	C6	C1	C10	120.1(2)
C68	C69	C88	128.16(17)	C10	C1	C2	107.9(2)
C70	C69	C88	125.45(16)	C3	C2	C1	119.9(2)
N2	C70	C69	111.66(15)	C3	C2	C12	120.1(2)
N2	C70	C71	124.36(16)	C12	C2	C1	108.2(2)

Atom	Atom	Atom	Angle/°
C2	C3	C4	119.9(2)
C2	C3	C15	120.0(2)
C15	C3	C4	108.1(2)
C3	C4	C17	107.92(19)
C5	C4	C3	120.1(2)
C5	C4	C17	120.0(2)
C4	C5	C6	119.9(2)
C4	C5	C20	119.4(2)
C6	C5	C20	108.7(2)
C1	C6	C5	120.2(2)
C1	C6	C7	119.9(2)
C5	C6	C7	107.6(2)
C8	C7	C6	119.8(2)
C8	C7	C21	120.2(2)
C21	C7	C6	107.9(2)
C7	C8	C9	120.3(2)
C7	C8	C24	120.0(2)
C24	C8	C9	107.7(2)
C10	C9	C8	119.8(2)
C10	C9	C26	120.0(2)
C26	C9	C8	108.28(19)
C1	C10	C11	108.0(2)
C9	C10	C1	120.1(2)
C9	C10	C11	119.7(2)
C12	C11	C10	108.10(19)
C28	C11	C10	120.2(2)
C28	C11	C12	119.8(2)
C2	C12	C11	107.83(19)
C13	C12	C2	119.9(2)
C13	C12	C11	120.2(2)
C12	C13	C14	120.0(2)
C12	C13	C30	119.9(2)
C14	C13	C30	108.20(19)
C13	C14	C32	107.91(19)
C15	C14	C13	120.3(2)
C15	C14	C32	119.9(2)
C3	C15	C16	108.12(19)
C14	C15	C3	119.8(2)
C14	C15	C16	120.1(2)
C15	C16	C17	107.8(2)
C34	C16	C15	119.7(2)
C34	C16	C17	120.4(2)
C4	C17	C16	108.1(2)
C18	C17	C4	120.2(2)
C18	C17	C16	119.6(2)
C17	C18	C19	119.8(2)
C17	C18	C36	119.9(2)
C36	C18	C19	108.5(2)
C18	C19	C38	107.5(2)
C20	C19	C18	120.0(2)
C20	C19	C38	120.6(2)
C19	C20	C5	120.5(2)
C19	C20	C21	120.2(2)
C21	C20	C5	107.4(2)
C20	C21	C7	108.4(2)
C22	C21	C7	120.1(2)
C22	C21	C20	119.4(2)
C21	C22	C23	119.4(2)
C21	C22	C39	120.8(2)
C39	C22	C23	107.8(2)
C24	C23	C22	120.2(2)
C24	C23	C41	120.2(2)
C41	C23	C22	107.9(2)
C8	C24	C25	108.1(2)
C23	C24	C8	120.1(2)
C23	C24	C25	119.7(2)
C26	C25	C24	108.16(19)
C43	C25	C24	120.5(2)
C43	C25	C26	119.73(19)
C25	C26	C9	107.81(19)
C27	C26	C9	120.2(2)
C27	C26	C25	119.8(2)
C26	C27	C28	119.7(2)
C26	C27	C45	120.48(19)
C45	C27	C28	107.98(18)
C11	C28	C27	120.1(2)
C11	C28	C29	120.2(2)
C29	C28	C27	107.81(19)
C30	C29	C28	120.1(2)
C30	C29	C46	120.3(2)
C46	C29	C28	108.10(18)
C29	C30	C13	119.84(19)
C29	C30	C31	119.68(19)
C31	C30	C13	108.07(18)
C30	C31	C32	108.04(18)
C48	C31	C30	119.99(19)
C48	C31	C32	119.7(2)
C31	C32	C14	107.78(19)
C33	C32	C14	119.94(19)
C33	C32	C31	120.2(2)
C32	C33	C34	120.2(2)
C32	C33	C50	120.1(2)
C34	C33	C50	108.0(2)
C16	C34	C33	120.3(2)
C16	C34	C35	119.8(2)
C35	C34	C33	108.0(2)
C34	C35	C51	108.0(2)
C36	C35	C34	119.9(2)
C36	C35	C51	120.3(2)
C18	C36	C37	107.7(2)
C35	C36	C18	120.3(2)
C35	C36	C37	119.9(2)
C38	C37	C36	108.1(2)
C53	C37	C36	119.6(2)
C53	C37	C38	120.7(2)
C37	C38	C19	108.2(2)
C39	C38	C19	119.3(2)
C39	C38	C37	119.8(2)
C22	C39	C40	108.5(2)
C38	C39	C22	119.8(2)
C38	C39	C40	119.8(2)
C39	C40	C41	107.5(2)
C54	C40	C39	120.0(2)
C54	C40	C41	119.9(2)
C23	C41	C40	108.3(2)
C42	C41	C23	120.3(2)
C42	C41	C40	119.5(2)
C41	C42	C43	119.7(2)
C41	C42	C56	120.4(2)
C56	C42	C43	108.01(18)
C25	C43	C42	119.65(19)
C25	C43	C44	120.62(19)
C42	C43	C44	107.68(19)
C45	C44	C43	119.43(19)

Atom	Atom	Atom	Angle/°
C45	C44	C57	119.99(19)
C57	C44	C43	108.15(18)
C27	C45	C46	108.08(18)
C44	C45	C27	119.98(19)
C44	C45	C46	120.1(2)
C29	C46	C45	108.02(18)
C47	C46	C29	119.97(19)
C47	C46	C45	119.94(19)
C46	C47	C48	119.93(19)
C46	C47	C58	120.29(19)
C48	C47	C58	107.80(19)
C31	C48	C47	120.1(2)
C31	C48	C49	120.1(2)
C47	C48	C49	108.03(18)
C48	C49	C59	108.01(19)
C50	C49	C48	120.1(2)
C50	C49	C59	120.1(2)
C49	C50	C33	119.9(2)
C49	C50	C51	119.9(2)
C51	C50	C33	107.9(2)
C50	C51	C35	108.0(2)
C52	C51	C35	119.5(2)
C52	C51	C50	120.2(2)
C51	C52	C53	120.2(2)
C51	C52	C60	119.9(2)
C53	C52	C60	108.2(2)
C37	C53	C52	120.5(2)
C37	C53	C54	119.4(2)
C52	C53	C54	108.2(2)
C40	C54	C53	120.2(2)
C40	C54	C55	120.3(2)

Atom	Atom	Atom	Angle/°
C55	C54	C53	107.9(2)
C54	C55	C60	107.9(2)
C56	C55	C54	120.1(2)
C56	C55	C60	119.6(2)
C55	C56	C42	119.9(2)
C55	C56	C57	119.9(2)
C57	C56	C42	108.19(19)
C56	C57	C44	107.97(18)
C58	C57	C44	120.13(19)
C58	C57	C56	120.4(2)
C57	C58	C47	119.6(2)
C57	C58	C59	120.0(2)
C59	C58	C47	108.10(18)
C58	C59	C49	108.06(19)
C60	C59	C49	120.1(2)
C60	C59	C58	119.9(2)
C52	C60	C55	107.8(2)
C59	C60	C52	119.8(2)
C59	C60	C55	120.2(2)
C99	C98	C103	119.6(2)
C98	C99	C100	120.2(2)
C99	C100	C101	120.1(2)
C102	C101	C100	119.7(2)
C103	C102	C101	120.0(2)
C102	C103	C98	120.3(2)
C105	C104	C109	120.2(2)
C106	C105	C104	120.0(2)
C105	C106	C107	120.0(2)
C108	C107	C106	120.2(2)
C109	C108	C107	119.7(2)
C108	C109	C104	119.9(2)

Table S6.6: Hydrogen Fractional Atomic Coordinates (×10[4]) and Equivalent Isotropic Displacement Parameters (Å^2×10^3). U_{eq} is defined as 1/3 of the trace of the orthogonalised U_{ij}.

Atom	x	y	z	U_{eq}
H66	1853.74	6430.57	8127.4	18
H71	3776.66	8676.97	10023.72	16
H76	7122.72	4997.79	10035.33	15
H81	5909.91	3389.29	7264.25	18
H82A	5114.82	3357.62	6270.79	24
H82B	4016.79	3629.68	6097.66	24
H83A	4439.64	2001.62	6406.45	44
H83B	3495.76	2547.52	7091.66	44
H83C	4603.73	2266.68	7249.48	44
H84A	2433.19	4624.78	6505.88	25
H84B	1907.48	5631.66	6972.82	25
H85A	869.57	4546.31	7356.57	48
H85B	1218.27	4821.41	8115.44	48
H85C	1754.67	3810.13	7657.22	48
H86A	938.86	7996.34	8220.61	21
H86B	661.79	8933.03	8782.65	21
H87A	-536.69	8019.16	9248.33	37
H87B	78.33	8031.01	9913.37	37
H87C	340.34	7103.31	9343.54	37
H88A	1487.56	9826.53	9346.96	22
H88B	2522.73	9773.23	9592.98	22
H89A	823.76	9209.48	10607.81	40
H89B	1164.51	10163.8	10736.37	40
H89C	1864.14	9131.36	10853.87	40
H90A	5293.53	8397.07	11453.18	19

Atom	x	y	z	U_{eq}
H90B	4502.74	8904.43	10946.71	19
H91A	5837.12	9287.88	9949.35	50
H91B	6619.18	8794.39	10468.23	50
H91C	5763.46	9747.5	10799.46	50
H92A	7057.26	5954.46	11098.12	19
H92B	6775.24	7070.67	11337.35	19
H93A	8169.62	6252.22	9878.94	32
H93B	8493.31	6575.05	10634.01	32
H93C	7895.12	7365.59	10129.07	32
H94A	8562.61	2697.08	9029.59	19
H94B	8087.88	3439.95	9771.97	19
H95A	9301.85	3869.03	8268.56	30
H95B	9680.41	3666.61	9076.65	30
H95C	8824.89	4614.15	9009.98	30
H96A	7151.11	2263.7	7546.3	23
H96B	7966.81	2020.43	8073.72	23
H97A	8766.56	2121.71	6704.33	43
H97B	8958.54	2930.23	7217.42	43
H97C	8132.78	3212.35	6699.6	43
H98	3095.13	5993.13	4205.02	32
H99	2271.66	7614.58	4426.22	34
H100	2313.67	8420.26	5559.63	36
H101	3191.39	7605.98	6472.86	38
H102	4100.57	6008.02	6208.29	41
H103	4011.43	5192.75	5099.16	39
H104	2836.59	9333.25	7096.41	37
H105	1164.42	9485.32	7072.44	32
H106	-59.94	10850.42	7617.47	39
H107	388.27	12072.35	8180.55	45
H108	2062.95	11923.95	8207.8	41
H109	3291.97	10555.23	7655.76	41
H61A	8087(17)	7543(16)	6613(15)	51
H61B	7460(20)	8593(13)	6626(15)	51
H61C	7800(20)	8061(19)	5833(10)	51
H61D	7015(15)	7788(19)	6499(16)	51

S7. Full references for those abbreviated in the main paper

[17] A. Krachmalnicoff, R. Bounds, S. Mamone, S. Alom, M. Concistrè, B. Meier, K. Kouřil, M. E. Light, M. R. Johnson, S. Rols, A. J. Horsewill, A. Shugai, U. Nagel, T. Rõõm, M. Carravetta, M. H. Levitt, R. J. Whitby, *Nat. Chem.* **2016**, *8*, 953-957.

[20] C. Beduz, M. Carravetta, J. Y.-C. Chen, M. Concistrè, M. Denning, M. Frunzi, A. J. Horsewill, O. G. Johannessen, R. Lawler, X. G. Lei, M. H. Levitt, Y. J. Li, S. Mamone, Y. Murata, U. Nagel, T. Nishida, J. Ollivier, S. Rols, T. Rõõm, R. Sarkar, N. J. Turro, Y. F. Yang, *Proc. Natl. Acad. Sci. USA* **2012**, *109*, 12894-12898.

[21] S. Mamone, M. Ge, D. Hüvonen, U. Nagel, A. Danquigny, F. Cuda, M. C. Grossel, Y. Murata, K. Komatsu, M. H. Levitt, T. Rõõm, M. Carravetta, *J. Chem. Phys.* **2009**, *130*, 081103.

[28] S. Bloodworth, J. Gräsvik, S. Alom, K. Kouřil, S. J. Elliott, N. J. Wells, A. J. Horsewill, S. Mamone, M. Jiménez-Ruiz, S. Rols, U. Nagel, T. Rõõm, M. H. Levitt, R. J. Whitby, *Chemphyschem* **2018**, *19*, 266-276.

[31] S. Mamone, M. Concistrè, E. Carignani, B. Meier, A. Krachmalnicoff, O. G. Johannessen, X. G. Lei, Y. J. Li, M. Denning, M. Carravetta, K. Goh, A. J. Horsewill, R. J. Whitby, M. H. Levitt, *J. Chem. Phys.* **2014**, *140*, 194306.

S8. References for the supporting information

[1] W. Adam, J. Bialas, L. A. Hadjiarapoglou, *Chem. Ber.*, **1991**, *124*, 2377-2377.

[2] D. W. Allen, B. G. Hutley, T. C. Rich, *J. Chem. Soc., Perkin Trans. 2*, **1973**, 820-822.

[3] K. Kurotobi, Y. Murata, *Science*, **2011**, *333*, 613-616.

[4] T. Futagoishi, M. Murata, A. Wakamiya, T Sasamori, Y. Murata, *Org. Lett.* **2013**, *15*, 2750-2753.

[5] S. Bloodworth, J. Gräsvik, S. Alom, K. Kouřil, S. J. Elliott, N. J. Wells, A. J. Horsewill, S. Mamone, M. Jiménez-Ruiz, S. Rols, U. Nagel, T. Rõõm, M. H. Levitt, R. J. Whitby, *Chemphyschem* **2018**, *19*, 266-276.

[6] A. Krachmalnicoff, M. H. Levitt, R. J. Whitby, *Chem. Commun.* **2014**, *50*, 13037-13040.

[7] C. Bengs, M. H. Levitt, *Magn. Reson. Chem.*, **2018**, *56*, 374-414.

[8] O. W. Sørensen, *Prog. Nucl. Magn. Reson. Spectrosc.*, **1989**, *21*, 503-569.

[9] H. M. Lee, M. M. Olmstead, T. Suetsuna, H. Shimotani, N. Dragoe, R. J. Cross, K. Kitazawa, A. L. Balch, *Chem Commun*, **2002**, 1352-1353.

[10] M. M. Olmstead, D. A. Costa, K. Maitra, B. C. Noll, S. L. Phillips, P. M. van Calcar, A. L. Balch, *J. Am. Chem. Soc.* **1999**, *121*, 7090-7097.

[11] O. V. Dolomanov, L. J. Bourhis, R. J. Gildea, J. A. K. Howard, H. Puschmann, *J. Appl. Cryst.*, **2009**, *42*, 339-341.

[12] G. M. Sheldrick, *Acta Cryst.*, **2015**, *C27*, 3-8.

S9. Author contributions

The project was conceived and coordinated by R.J.W and M.H.L. The manuscript was written by S.B, with contributions from R.J.W and M.H.L. Synthesis of all new compounds was carried out by S.B and purification of $CH_4@C_{60}$ was carried out by G.S. S.V assisted in initial high-pressure filling studies and provision of additional material. Synthesis of a precursor to the compounds reported in this article was carried out by S.A using the published methods. Mass spectrometric analysis of $CH_4@C_{60}$ was carried out by J.M.H and G.J.L, and NMR analysis of $CH_4@C_{60}$ was carried out by G.R.B and S.J.E. The crystal structure was acquired and solved by M.E.L.
